# Neuromuscular Performance of High-Level Football Goalkeepers by Age Category and Sex: A Systematic Review

**DOI:** 10.3390/jfmk10040398

**Published:** 2025-10-13

**Authors:** Pablo González-Jarrín, Jaime Fernández-Fernández, José Vicente García-Tormo, Carlos Gutiérrez García

**Affiliations:** Faculty of Physical Activity and Sports Sciences, Universidad de León, 24071 León, Spain; pablogonzalezjarrin@gmail.com (P.G.-J.); jvgart@unileon.es (J.V.G.-T.); cgutg@unileon.es (C.G.G.)

**Keywords:** soccer, athletic performance, physical fitness assessment, age groups, sex differences

## Abstract

**Background:** Goalkeeper actions directly influence match outcomes and overall team performance. Neuromuscular determinants (e.g., perception–action coupling, reaction speed, rate of force development (RFD), balance, etc.) translate into higher save probability, faster second actions, and more accurate distribution. **Objectives:** This systematic review analysed neuromuscular performance factors in 11-a-side football goalkeepers and examined how these factors evolve across age and sex groups. **Methods:** The review adhered to the PRISMA 2020 Statement guidelines. A systematic search was conducted using the PubMed, Web of Science, SPORTDiscus, PsycINFO, Dialnet, LILACS, and Scopus databases. Studies that assessed any aspect of goalkeepers’ neuromuscular performance except for aerobic endurance and VO_2_ max (due to the short duration of goalkeeping actions) were included, regardless of the type of observational design. **Results:** Thirty-five studies were finally included in the synthesis, encompassing neuromuscular performance factors such as agility, speed, anaerobic power, strength, flexibility, and dynamic balance and coordination. The findings underscore the need for neuromuscular training for goalkeepers, particularly agility training. Neuromuscular performance improves with age, especially in linear speed, agility, change-of-direction speed, strength, and power; however, flexibility shows no significant progression. This review identifies key tests for evaluating goalkeepers’ neuromuscular capacities across major performance domains. **Conclusions:** Although sex differences are apparent, the main limitation is the lack of research on neuromuscular performance in male and female goalkeepers, making it difficult to define indicators for different age and sex categories.

## 1. Introduction

Football is a sport characterised by its tactical complexity and dynamism [1,2]. The primary goal of a football match is to win, which can be achieved in two ways: by preventing goals and scoring one, or by scoring one more than the opponent. Teams have only ~60–74 min with the ball-in-play [3], with intermittent, predominantly anaerobic efforts influenced by situational and contextual variability [4,5]. This highlights the critical role of both aerobic and anaerobic endurance in football performance.

The physical demands of football vary considerably depending on a player’s position on the pitch, with goalkeepers showing the most marked differences between positions. Outfield players typically cover a total distance of between 10,787.9 ± 1536.8 and 9272.5 ± 455.7 m per match [6], while goalkeepers cover about half that distance, between 5611 ± 613 and 4084.13 ± 577 m [6,7,8,9,10,11]. Outfield players cover between 1233 ± 360 and 1683 ± 252 m per match at high intensity (18 km/h) [3], while goalkeepers cover between 221 ± 90 and 230 ± 108 m per match at these speeds (14.5–20 km/h) [12,13]. While this information may suggest that goalkeepers are less physically demanding than their outfield counterparts, it should prompt physical trainers and goalkeeper coaches to consider whether goalkeepers require similar training and recovery strategies to players in other positions.

Nevertheless, the physical effort required of goalkeepers is far from trivial, given their pivotal role in preventing goals and influencing the outcome of matches [11]. Goalkeepers are involved in direct interventions approximately every 2.5 ± 0.3 min and perform an average of 36 ± 4 actions per match, 18 ± 4 of which occur in each half [10]. During the match, they perform approximately 8 ± 3 changes in direction [11]. During forward transitions they typically cover distances of 17.5 ± 7.56 m, and during lateral changes 15.5 ± 7.78 m [14]. Defensive interventions result in an average of approximately 3.74 to 4.99 saves per match in various top European leagues (i.e., Premier League, La Liga, Bundesliga, Serie A) [15]. Despite their traditional defensive role, goalkeepers are also actively involved in offensive play [11], making between 25.8 and 36.5 offensive interventions per game in the aforementioned top European leagues [15]. Overall, goalkeepers’ actions are characterised by short intermittent periods of maximal intensity, which should be able to adapt in space and time [16]. From a physiological point of view, these short and intense actions rely primarily on the anaerobic rather than the aerobic energy system [17].

In recent years, there has been a noticeable increase in the number of studies focusing on the physical performance of goalkeepers. However, only a few comprehensive reviews have been published. Ziv and Lidor’s [18] quasi-systematic review analysed the physical characteristics, physiological attributes, and on-field performance of football goalkeepers. They found that goalkeepers experienced fatigue earlier than players in other positions, probably due to lower aerobic capacity. Furthermore, the vertical jump performance of goalkeepers was higher than that of the rest of the team, which can be considered a crucial attribute for goalkeepers. West [19] conducted a narrative review to identify factors influencing goalkeeper performance, covering physiological, tactical, technical (both defensive and offensive), and psychological aspects. This review highlighted the importance of parameters such as anthropometry, strength, power, agility training, as well as specific movements such as blocks, detours, and various accelerations and decelerations. However, the lack of methodological information makes it difficult to assess the quality of this review. White et al. [20] developed another narrative review that focused on goalkeeper performance during match play, covering both physical and technical aspects. They also examined individual performance tests focusing on strength, power, speed, aerobic capacity, football specific skills, and other relevant factors. Their analysis argued that goalkeepers have a unique profile compared to outfield players, with different physical and mental demands, and often lower aerobic capacity (i.e., maximum oxygen uptake—VO2max). It is worth noting that their sole reliance on the PubMed database for document searches may have limited the scope of their findings [21]. Finally, Pérez-Arroniz et al. [22] conducted a systematic review analysing various components of goalkeeper fitness, including anthropometric measures, conditional attributes (such as sprinting, jumping, agility, strength, and mobility), external load, and injury profiles. Their findings emphasised the critical role of sprinting, jumping, agility, and mobility in determining goalkeeper performance, while suggesting that aerobic capacity may be less important. For example, the review compiles test outcomes with countermovement-jump heights typically around~40–48 cm and short-sprint (≤10 m) times near ~1.1–1.9 s, reinforcing the primacy of explosive and anaerobic capacities over aerobic endurance. In addition, this review highlighted worrying trends in the training practices of goalkeepers aged 9–13 years, which shed light on potential areas for improvement in player development strategies. It should be noted that although the authors state that this review was conducted in accordance with the PRISMA statement [23], it lacks important elements such as the assessment of risk of bias.

All of these previous reviews agree that goalkeepers have a unique external load during competition compared to outfield players, underpinned by distinctive physical and mental attributes. In particular, goalkeepers excel in sprinting, jumping, agility, and mobility, highlighting the critical role these qualities play in their performance [1,22,24]. For example, professional goalkeepers exhibited higher countermovement-jump performance than defenders and midfielders (CMJ 41.9 ± 2.1 cm vs. 36.8 ± 3.0 cm and 38.0 ± 3.3 cm, respectively) and were faster in number- and shape-based choice reaction time tasks [24]. While aerobic endurance may not be as important, previous research suggests that agility is the linchpin of goalkeeping effectiveness. Quick reactions to shots, accurate throws, and rapid changes in direction underline the importance of agility in their game [24].

In this context, the neuromuscular system plays a crucial role in orchestrating muscle activation, body adaptation, and balance maintenance in response to stimuli. In addition to the physical capacities such as strength, speed, and endurance highlighted in the review by Pérez-Arróniz [22], additional qualities such as flexibility, balance, stability, and coordination need to be considered. Therefore, while acknowledging the breadth of topics covered in other reviews, such as psychological, anthropometric, technical–tactical, and injury profiles, the identification of key performance factors specific to goalkeepers is still under debate. Therefore, the aim of the present review is to analyse neuromuscular performance factors (i.e., agility, speed, strength, anaerobic power, mobility, balance, and coordination) in goalkeepers. In addition, we aim to investigate the development of neuromuscular performance in goalkeepers across different age groups and genders in 11-a-side football. This research is particularly relevant in light of the worrying training trends in young goalkeepers (aged between 9 and 13) highlighted in the review by Pérez-Arróniz [22].

## 2. Materials and Methods

This systematic review followed the Preferred Reporting Items for Systematic Reviews and Meta-analyses Protocols (PRISMA-P) statement [25], and the review protocol was registered on the Open Science Framework Registries—OSF [26]; (Registration number: DOI 10.17605/OSF.IO/ZWN3D).

### 2.1. Eligibility Criteria

Specific studies on 11-a-side goalkeepers were included in this review, regardless of age and sex. Studies involving 11-a-side players in different positions were also included if they provided specific data on goalkeeper performance. Studies that included goalkeepers from similar sports (e.g., indoor football) were excluded. These studies had to focus on any expression of neuromuscular performance (i.e., agility, speed, strength, anaerobic power, mobility, balance, and coordination) that characterised goalkeeper actions, such as sprinting, jumping, diving saves, one-on-one actions, and other movements. However, as these actions typically last between 5 and 20 s, they were primarily influenced by anaerobic power. Therefore, we decided not to include aerobic endurance and VO2max, as these were not significant performance factors for goalkeepers [18,20,22].

This research focused on high-level goalkeepers to provide clear neuromuscular performance indicators. High-level status was defined as playing in the top two divisions of a country or representing the national team. However, in World Cup-winning countries with highly professionalised leagues, even fourth division players maintained elite levels of demand and performance. Previous research had highlighted the development of talent within competitive systems, where lower division players often have experience at the highest level [27].

On the other hand, we decided not to include studies that focused specifically on penalty kicks. Although penalty kicks place similar neuromuscular demands on the goalkeeper as other actions, actual performance was often strongly influenced by other factors, such as prior study of the shooter’s kicking patterns or the psychological strategies were used by goalkeepers to interfere with the shooter [28]. In addition, studies not related to the aim of the study were excluded, such as those on injuries, goalkeeper-specific equipment, external loads, training methods, technical analysis, small-sided games, psychological and sociological aspects, technical–tactical actions, robots, or anthropometry.

Following the classification of Grimes and Schulz [29], all types of observational studies were included. Studies had to be original and published in full length as a journal article, book, or book chapter. Therefore, duplicate publications, short communications, or abstracts published in conference proceedings were excluded. Doctoral theses were also excluded. Only studies published in English, Portuguese, and Spanish were included, as these were the languages known to the authors. Finally, only articles published from 1997 onwards were included, as this was the year in which the current regulations for professional football were consolidated. These regulations stipulated that the goalkeeper is allowed to hold the ball for a maximum of six seconds [30], which meant that more effective time is played and more actions are developed by the goalkeeper.

### 2.2. Information Sources

The literature search followed PRISMA-S, the extension of the PRISMA statement for reporting literature searches in systematic reviews [31]. Several multidisciplinary and specific databases were searched for documents. Multidisciplinary databases included Scopus and the Web of Science Core Collection. Specific databases included PubMed (PubMed), Sport Discus (EBSCOHost), PsycINFO (EBSCOHost), PsycBOOKS (EBSCOHost), PsycARTICLES (EBSCOHost), and Psychology and Behavioral Sciences Collection (EBSCOHost). Regional databases such as the Health Sciences in Latin America and the Caribbean—LILACS (LILACS), the Scientific Electronic Library Online—SciELO (Web of Science), and Dialnet (DIALNET) were also searched, as this strategy had been shown to retrieve the relevant literature for inclusion in systematic reviews [32,33]. The Web of Science Core Collection and SciELO were searched simultaneously via the Web of Science platform, and PsycINFO, PsycBOOKS, PsycARTICLES, and the Psychology and Behavioral Sciences Collection were searched via EBSCOHost. Search alerts were then set in all databases where this option was available. Backward and forward snowballing techniques [34] were used to identify additional studies. This was performed by manually screening the reference lists of the included articles and using the citation tools provided by several databases such as Scopus, Web of Science, or PubMed. It was not necessary to contact authors or experts to find additional data.

### 2.3. Search Strategy

The research team tested several terms (e.g., football, soccer, goalkeeper, performance, neuromuscular performance, strength, muscle power, speed, resistance, balance, etc.) to create an optimal search string. Due to the variety of terms used in the literature on this topic, it was decided to perform a broad search that would minimise the loss of potentially eligible studies. Therefore, only the terms ‘football’, ‘soccer’ and ‘goalkeeper’ were considered. Unique search strings were constructed depending on the operators accepted by each database (e.g., Boolean operators, wildcards, truncation symbols). The search strings for the different databases were as follows:

- PsycINFO, PsycBOOKS, PsycARTICLES, Psychology and Behavioral Sciences Collection, Dialnet, SPORTDiscus, and PubMed: (Football* OR soccer*) AND goalkeeper*
- LILACS, Scopus: (Football* OR soccer*) AND goalkeeper*, with filter (title/abstract/subject)
- Web of Science: (Football* OR soccer*) (topic) AND goalkeeper* (topic)

The first database search was conducted on 20 January 2024, following the doctoral research developed by the first author. We searched all databases where this option was available (Scopus, Web of Science, PubMed, PsycINFO, PsycBOOKS, PsycARTICLES, Psychology and Behavioral Sciences Collection, Dialnet, SportDiscus, Lilacs) and set citation alerts. A final search of all databases was conducted on 30 March 2025, using the same strategy and the same databases as the initial search in order to include the most recent studies available.

### 2.4. Selection and Data Collection Process

**Figure 1:**
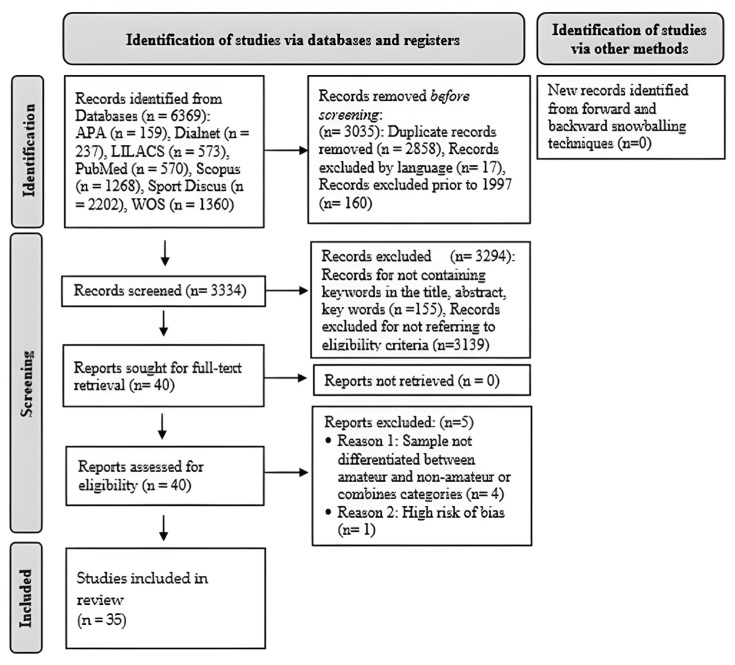
Flow diagram of the review process. Based on the PRISMA-P recommendations [23]. Note: PRISMA-P = Preferred Reporting Items for Systematic Review and Meta-Analysis Protocols; APA = American Psychological Association; WOS = Web of Science.

The selection and data collection process consisted of three main phases (see the review flowchart in Figure 1). In the identification phase (phase 1), all basic information (authors, titles, sources, DOIs, abstracts, keywords, etc.) of the retrieved documents was exported from the selected databases into the EndNote (version.X9; Clarivate Analytics, Philadelphia, PA, USA) reference management software, without applying any additional restrictions to the search string. Duplicate records were then automatically eliminated using EndNote’s ‘find duplicates’ search tool, followed by a manual search to discard duplicate documents not detected by the automated search tool. Records published before 1997, and records not published in English, Spanish, or Portuguese were then excluded. We also used forward and backward snowballing techniques to look for additional documents to include in the review.

Phase 2—screening, consisted of 3 steps. In step 1, record selection, the title, abstract, and keywords of the records selected from phase 1 were screened against the eligibility criteria. In step 2, full text retrieval, the full text of the remaining records was retrieved. In step 3, eligibility, a detailed assessment of the potentially eligible studies was carried out, including a reassessment of the eligibility criteria and risk of bias assessment.

Finally, in step 3, inclusion, a standardised form was used to extract and synthesise data from the selected studies (see below, section on data items and outcomes).

All these stages were carried out independently by two members of the review team. A third member of the team helped to resolve any discrepancies or disagreements.

### 2.5. Data Items and Outcomes

This review considered the following data elements: type of study, study aims, sample, instrument, surfaces, measurements, and outcomes related to goalkeeper performance.

The outcomes of interest in this review were the neuromuscular performance values of goalkeepers and the differences in neuromuscular performance between goalkeepers of different categories and sex. In addition, the methodological procedures followed in the studies published on this topic were assessed.

### 2.6. Study Risk of Bias Assessment

All selected studies were descriptive or analytical cross-sectional studies. Following Ma et al. [35], two tools were used at this stage: the JBI Critical Appraisal Checklist for Analytical Cross-Sectional Study [36] was used to assess analytical cross-sectional studies, and the JBI Critical Appraisal Checklist for Studies Reporting Prevalence Data [37] was used to assess descriptive studies. Two members of the review team independently assessed the quality of the selected studies, and a third member helped to resolve disagreements between the two main reviewers.

### 2.7. Synthesis Methods

The findings of this review are presented in a narrative synthesis. We also provided summary tables of the methodological quality assessment and the main characteristics for each study.

## 3. Results

### 3.1. Study Selection

Figure 1 shows the flowchart for the present review. In phase 1, identification, 6369 registries were retrieved, 3294 of which were discarded because they were duplicated, published before 1997, or were not published in English, Spanish, or Portuguese. In phase 2, screening, the remaining 3334 records were checked against eligibility criteria, discarding a total of 2962 registries for the reasons shown in Figure 1. Then, the full text for the 40 remaining studies was screened against the same eligibility criteria and risk of bias. Four studies [38,39,40,41] were excluded as they did not meet the inclusion criteria and one [42] was excluded due to high risk of bias. The identification of studies via other methods (forward and backward snowballing techniques) did not result in any new study being included. Finally, 35 studies were included in the review.

### 3.2. Study Characteristics

A total of 35 studies were selected for review, including four analytical cross-sectional studies and 31 purely cross-sectional studies. Each study is described in Table A1 (See Appendix A), including study type, study objectives, sample, procedures, measurement, and results.

### 3.3. Risk of Bias Assessment

Table 1 showed the results of the review of the four analytical cross-sectional studies included in the review. The studies clearly defined the criteria for selecting the sample and how the condition was measured. While more detailed descriptions may be needed in some cases, the subjects and study environment are generally well characterised. Both exposures and outcomes were measured in a valid and reliable way, with appropriate statistical analysis. However, it is clear that the studies should place greater emphasis on strategies to identify and control confounding variables.

Table 2 showed the results of the risk of bias assessment for the remaining 32 purely cross-sectional studies. For both assessments, we excluded studies where more than half of the questions in each tool were answered negative. As a result, the study by Pivovarniček et al. [42] was excluded as it contained four negative responses and one “unclear” answer out of nine questions.

The main problem identified in the chosen cross-sectional studies was that the sample size was often not big enough. However, the sample selection is generally well aligned with the target population. Most studies provided a detailed description of the subjects and settings, and a clear explanation of how the data was analysed. Assessments were conducted consistently and reliably across all participants, resulting in an adequate response rate. However, in some cases, the methods used were not good enough to accurately identify the condition, and certain studies lacked transparency in reporting the statistical analyses conducted.

### 3.4. Findings

#### 3.4.1. Results of Individual Studies

Table A1 (see Appendix A) summarised the key findings of each study, according to the studied variables. When multiple sample groups were included, the corresponding data are detailed for each group.

#### 3.4.2. Results of the Syntheses

This review focused on the football goalkeeper. The main challenge influencing the risk of bias is the difficulty in finding studies with a substantial number of goalkeepers. Additionally, a potential source of bias arose from the lack of strategies for identifying and mitigating confounding factors. It was also noted that some studies failed to clearly define the testing environment, meaning that surface conditions could influence performance outcomes. Furthermore, statistical data should be presented more visually, as not all reported statistical analyses were fully displayed. Please refer to Table 1 and Table 2 for a comprehensive overview of the risk of bias in each study.

There was considerable heterogeneity among studies due to variations in the measured variables, statistical significance levels, and effect sizes. Additionally, the limited statistical information provided in some studies made it difficult to determine the exact extent of this heterogeneity with certainty (see Table A1). The table also highlighted statistical robustness and key data extracted from the studies.

##### Agility

The goalkeeper responded to the opposing team’s attacks by perceiving stimuli and making tactical decisions, either “reasoned” or “automatic,” based on time and attack countering. This process began when sensory information is transmitted from receptors, such as the eyes, to the spinal cord or brain. The brain then generated motor orders to the muscles [76,77]. Agility, defined as “a rapid movement of the whole body with a change in speed or direction in response to a stimulus” [77], is therefore the most crucial physical attribute for a goalkeeper.

Based on this definition, we have identified two agility tests: the RAS (Reaction and Action Speed) test and the G-RAT (Goalkeeper-specific Reactive Agility Test). In the simple version of RAS test, the goalkeeper reacted to a panel displaying one of four illuminated LEDs, with each LED corresponding to a corner of the goal. Upon activation, the goalkeeper had to dive to the indicated corner to intercept the ball [43]. The complex RAS test added an additional challenge: after responding to the initial LED by diving to the top corner, the goalkeeper had to quickly transition to the opposite bottom corner. The results of these tests and their implications for goalkeeping performance are summarised below in Table 3.

In the G-RAT, goalkeepers started at the centre of the goal and moved forward. At a distance of 1.5 m, they received an audible signal (A, B, C, or D) as they approached a central point 3.5 m from the start. They then had to retrieve the ball that has been placed on the ground in the designated zone (A, B, C, or D). Zones A and D required a 90° change in direction, while zones B and C involved a 45° change. A study of 9 starting and 24 substitute goalkeepers found that “G-RAT without diving” times averaged 11.7 ± 0.6 s for starters and 12.1 ± 0.7 s for substitutes, while “with diving” times were 14.3 ± 0.7 s and 15.3 ± 1.0 s, respectively [78]. One limitation of the test is that it did not account for directionality or turning angles, grouping the results without distinguishing that 90º turns cause more deceleration than 45° turns.

Two years later, the authors of the G-RAT developed an adapted version for adolescents: the Goalkeeper Reactive Agility Test for Adolescents (GRATA), in which the stimulus changed from auditory to visual [79]. Sub-14 goalkeepers achieved a time of 11.98 ± 0.87 s, maintaining the previous limitation of the G-RAT. The test demonstrated sufficient reliability and content validity within this population. However, this study was not selected due to the lack of data on high-level goalkeepers.

##### Velocity

Speed was a crucial physical attribute for football goalkeepers, encompassing various components that directly impact their performance: reaction and gesture speed, velocity in linear movements, and movement speed with direction changes.

(a) Reaction and gesture speed

As the final line of defence, goalkeepers had to react instantly to the opponent’s actions [15,22,80], processed stimuli, and made positioning decisions before executing a technical save [19,24]. However, there was a lack of research in this area.

(b) Velocity in linear movements

Linear speed was a frequently evaluated parameter in football. In this review, sprint tests over 10, 20, and 30 m were applied more frequently than longer-distance tests. A total of 15 studies analysing 246 goalkeepers of different ages and performance levels were reviewed (see Table 4).

Linear speed is a frequently evaluated parameter in football. In this review, sprint tests over 10, 20, and 30 m were applied more frequently than longer-distance tests. A total of 15 studies analysing 246 goalkeepers of different ages and performance levels were reviewed (see Table 4).

(c) Movement Speed with Direction Changes

As previously defined, the term ‘agility’ was frequently misused in tests designed to assess quick, multidirectional movements in the absence of stimuli. These tests evaluated maximum multidirectional speed without incorporating decision-making processes. In this review, two goalkeeper-specific tests were identified: the Sprint-Keeper (S-Keeper) and the Lateral Shuffle-Keeper (LS-Keeper). However, these studies were excluded from the main analysis as their samples were limited to goalkeepers aged 11 to 18, restricting their applicability to other categories. The S-Keeper assessed a goalkeeper’s ability to move forward, change direction, and dive for a low ball without an external stimulus, while the LS-Keeper incorporated lateral movements [38].

A total of seven studies analysing 131 goalkeepers of different ages and skill levels were reviewed. The most commonly used test was the *t*-test, implemented in two studies (Table 5) with a sample of 100 goalkeepers from three categories. However, other tests had not been investigated in age- or sex-differentiated samples. Some reported values included: Male U16: (5-0-5 test (dominant = 2.59 ± 0.11 s; non-dominant = 2.61 ± 0.23 s); K-test = 10.85 ± 0.4 s [61]). Male O19: (Four-line sprint test (14.19 ± 0.26 s) [48]; 10–5 m (0.76 ± 0.06 s) and shuttle run (SR) (12.32 ± 0.44 s [17]). Women O19: (Pro-agility shuttle = 4.93 ± 0.0 s; Arrowhead test (9.20 ± 0.0 s on both sides) [47], aligning with 9.18 ± 0.9 s [66]; 12.5 m agility test (Left = 10.50 ± 0.39 s; Right = 10.51 ± 0.41 s) [69]).

##### Anaerobic Power

The endurance of goalkeepers was linked to their anaerobic power, due to the brevity and high intensity of their interventions [16], without generating significant lactate levels. While research on this topic was limited when compared to studies of aerobic endurance [17,47,51,56,58,59,60,61,63,66] and lactic anaerobic endurance [1,56,59,61], it was crucial that goalkeepers could repeat these actions to delay the onset of peripheral fatigue. If fatigue occurred, maximal force and rate of force development declined, potentially impairing explosive actions, reaction time, and coordination, increasing time-to-take-off and reducing distribution accuracy [81]. When load was well managed, force output and contraction efficiency were maintained across repeated actions, making pre-match load management key to sustaining high-force performance and delaying fatigue [24].

##### Strength

A variety of tests were available to assess strength from different perspectives. This review identified ten studies involving a total of 302 goalkeepers from different ages and competition levels. The majority of the data were derived from tests that do not replicate in-game movements.

Although the Wingate test is related to the assessment of anaerobic capacity, it is not alactic, which made it more relevant to anaerobic power evaluation. In this test, goalkeepers pedal at maximum intensity for 30 s with a resistance determined by their body weight. It also allowed for the assessment of the fatigue index, which was only observed at 41.32 ± 5.88% [71]. In three studies involving 95 goalkeepers, it was observed that maximal, average, and relative maximum power increased with age, although this may not apply to average relative power (see Table 6).

A variety of tests were available to assess strength from different perspectives. The majority of the data were derived from tests that do not replicate in-game movements. In two studies involving 99 goalkeepers, an increase in strength values was observed in the force–velocity test performed on a cycle ergometer (see Table 7). Additionally, there was a discernible evolution in isometric trunk strength and its correlation with the legs.

A total of five studies involving 108 goalkeepers were conducted to assess the isokinetic strength of the leg flexor and extensor muscles. However, discrepancies were found in the measurement units used: degrees [1], degrees or milliseconds [74], and predominantly peak torque (Nm) assigned to degrees per second (°/s) (see Table 8). The studies’ primary findings suggested that the dominant or right leg demonstrates increased power, and when analysing movement speed (ranging from 30°/s to 240°/s), a consistent decline in torque was observed across all samples. Additionally, Ruas et al. [72] examined goalkeepers from the Brazilian Southern First Division, assessing movement from 0° to 90°, and reported peak torque values reflecting asymmetries in muscle power output: extension—Dom: 299.5 ± 30.6 Nm; NDom: 277.9 ± 33.3 Nm; flexion—Dom: 173.8 ± 33.1 Nm; NDom: 150.8 ± 31.5 Nm.

Goalkeepers relied on explosive power for most of their actions, including jumps, changes in direction, and saves. This was studied in 21 studies involving a sample of 376 goalkeepers. The primary findings indicated that male goalkeepers tended to exhibit greater jump height as they progress through categories and age groups (see Table 9). In contrast, with some exceptions, female goalkeepers over the age of 19 appeared to demonstrate performance levels comparable to those of male goalkeepers in lower categories. However, further research was needed to confirm this.

##### Flexibility

Flexibility has been the subject of study in five studies, and four on ROM have examined 199 goalkeepers across all categories and 60 over 19 years old, respectively. The sit-and-reach test values have showed consistency, with the exception of higher scores observed in Costa Rican goalkeepers [49] (Table 10).

The results showed greater ankle dorsiflexion and hip internal rotation in players from the Spanish Football Federation [52] (Table 11). Additionally, passive hip flexion and extension were similar between legs.

##### Dynamic Balance and Coordination

In this review, only one study was identified that assessed goalkeeper coordination as a predictor of talent [82]. Six specific tests were designed and administered to assess both general coordination and hand–eye coordination, with overall good reliability, although some individual tests showed lower coefficients. The results indicated that goalkeepers who scored higher on these tests were the most talented. However, this study was not selected due to the heterogeneity of the sample, as it included goalkeepers with different skill levels, which could affect the validity of the comparisons.

Goalkeepers outperformed other players in terms of balance, strength, and power [1], but might deliberately lose their balance when diving. Three studies of 34 goalkeepers found that stability improved with age, particularly in the posteromedial phase (see Table 12). The Y-balance test showed greater stability in the non-dominant leg, probably due to its role in passing and goal kicking. This suggested that experience and footwork training could improve the stability of the dominant leg. Goalkeepers excelled in posteromedial and medial stability, but had less control in anterior, anterolateral, and lateral directions, which could have affected their defensive performance.

#### 3.4.3. Reporting Biases

When evaluating reporting biases, we found that most studies presented the results consistently. However, the small sample size of some studies, along with the variability in the presentation of data and statistical analyses, could potentially introduce some bias (see Table 1 and Table 2).

#### 3.4.4. Certainty of Evidence

As with any systematic review, the certainty of the evidence depended on the number of available studies, their methodological quality, and the consistency of their findings. The most strongly supported physical capacities were linear speed and vertical jump performance, both assessed in multiple studies using similar methods. This allowed them to be classified as having high certainty, with a few methodological exceptions [45,55,62,68]. Other capacities—such as strength, change-of-direction speed, and flexibility (sit-and-reach test)—were evaluated in only a small number of studies. Despite this limitation, the similarity in outcomes supported a moderate level of certainty, although sample characteristics might have influenced some particularly strong performances. Finally, agility, mobility, and postural stability were associated with low certainty due to the scarcity of studies, heterogeneity in testing protocols, and lack of repeated measures across studies.

## 4. Discussion

This discussion critically interpreted the findings on goalkeepers’ physical performance, contrasting them with prior literature and the competitive context. It examined patterns by age, category, and sex, with attention to positional specificity and potential methodological biases. Practical implications for assessment and training were considered (e.g., test selection and priorities). Finally, gaps in the evidence and avenues for future research were identified to refine these conclusions.

### 4.1. Agility

The results of the RAS test show a clear improvement in agility with age (see Table 3). In addition, we observed that starting goalkeepers consistently outperformed substitutes in terms of agility in both the RAS and G-RAT tests, reflecting a strong correlation between agility and goalkeeper performance. Despite the importance of agility, it is important to note that it has not been assessed across all categories, nor has it been assessed in female goalkeepers. Furthermore, due to its specificity to goalkeepers, it has not been assessed in players from other positions. Rather than relying on pre-scripted, technique-only goalkeeper drills (e.g., predetermined chest catches or fixed lateral saves), coaches should emphasise agility-based tasks that couple perception and action under representative, variable conditions. Sessions ought to include unpredictable stimuli and evolving affordances—e.g., recovery runs with footwork adjustment to re-establish the bisector, rapid re-positioning relative to the ball carrier, and immediate reactions to (i) shots to near/far post or (ii) passes to a teammate—so that no two sequences are identical. This approach elicits repeated decisions (position, timing, technique selection) at match-like intensities, thereby targeting the neuromuscular and cognitive determinants of agility that differentiate starters from substitutes. Alternatively, prescribe a simple RAS block: 2–3 sets × 6–8 reps, one maximal dive per cue, ~2–3 s of work with 20–40 s of recovery. Future research should investigate goalkeeper agility in relation to category and sex.

### 4.2. Velocity

#### 4.2.1. Reaction and Gesture Speed

It has been observed that, for reaction speed and gestural responses, visual reaction time (RT) and stimulus analysis are key. However, reaction speed alone is insufficient without rapid movement speed and high rate of force development (RFD) to effectively intercept the ball [24]. Despite their importance, no studies met our inclusion criteria that isolated and quantified these components in goalkeepers. Future work should assess visual RT, movement speed, and RFD jointly and in isolation—using task-representative protocols—and report results stratified by competitive category and sex.

#### 4.2.2. Velocity in Linear Movements

Linear speed is crucial for goalkeepers as it allows them to reach optimal positions and improve their actions. Goalkeepers perform between 1.34 and 1.66 sprints per match [9], covering distances between 11 and 33 m [10,18,83]. Based on this, and the frequency of testing in research, it is recommended to assess linear speed using the 30-m sprint, with partial times recorded at 10 and 20 m.

Acceleration at 5 and 10 m generally improves with age for male goalkeepers, although there are inconsistencies in some samples (see Table 4). In the 5 m sprint, progress continues for Turkish Super League goalkeepers [60], but not for Croatian League goalkeepers [55] or Belgian League goalkeepers [17]. In the 10-m sprint, performance stagnates in the U19 Tunisian first division [68] and the U19 Croatian First National League [56]. Compared with other positions, these values tend to converge, especially at 5 m [17,54,55,56,59,60,61], although it is difficult to reach statistical significance.

Unlike the 30-m sprint, performance in the 20-m sprint does not improve consistently with age. In the 20 m sprint, nine U-16 goalkeepers from the Czech first division [61] outperformed both U-19 and senior goalkeepers. In the 30 m sprint, however, only goalkeepers from the Tunisian first division performed worse [68]. As the distance increases, the difference between goalkeepers and other positions increases [49,50,51,55,61,65,68], with goalkeepers also performing below average.

On the other hand, female goalkeepers performed similarly to male U14 goalkeepers in the 5 m and 10 m sprints [47], with times both above and below those of male U19 goalkeepers. In the 20 m sprint, however, they were equal to or better than male goalkeepers over 19, but were inferior in the 30 m sprint. It is worth noting that as the sprint distance increases, speed measurements become less common. Other results show that 14 female goalkeepers aged 19 and over from the Norwegian Women’s Premier League covered 40 m in 5.92 ± 0.28 s [69], while male goalkeepers aged 19 and over covered 60 m in 7.65 ± 0.19 s [65]. This highlights a potential strength in short-distance acceleration, which is particularly relevant to the demands of the goalkeeper position. When compared to other positions on the pitch, female goalkeepers show mixed results. While they perform worse than other positions [47,69], they also perform better than defenders, midfielders, and attackers [58,66]. Future studies should further explore these differences, taking into account positional roles, training exposure, and the influence of competition level and sex on sprint performance.

#### 4.2.3. Movement Velocity with Changes in Direction

According to the previous definition, the term “agility” is often misused in tests designed to assess fast, multidirectional movement without any stimulus. These tests measure maximum multidirectional speed with no decision-making involved. Goalkeepers’ movements are highly multidirectional, with an average of 8 ± 3 directional changes per match [11], 12 ± 0.46 m of lateral step [84], and 40 ± 28.2 lateral movements, with a lateral step advancing 3.70 ± 2.12 m [14].

Among the less specific tests for goalkeepers, the *t*-test stands out (Table 5). It shows that elite Belgian goalkeepers improve with age and that elite Portuguese U19 goalkeepers do not outperform elite Belgian goalkeepers [50,51]. Meanwhile, non-elite goalkeepers (over 19 years old) perform worse than elite goalkeepers from Belgium and Portugal. In addition, two goalkeeper-specific tests were identified: Sprint-Keeper and Lateral Shuffle-Keeper [38]. Although these tests include goalkeepers aged between 11 and 18, they are specifically designed for goalkeepers, which limits their applicability to goalkeepers of other ages or skill levels. Therefore, the *t*-test is recommended for assessing speed and change in direction, while the LS and S-Keeper tests can be alternatives for position-specific assessments. However, these tests have not been sufficiently investigated across different age and sex groups, suggesting that future research should investigate speed and direction changes in samples stratified by age and sex.

When comparing these tests with samples from other positions, goalkeepers showed lower performance than other positions and the average of all categories in the *t*-test [50,51]. As we can see, male U16 goalkeepers had lower scores than all other positions on both sides of the 5-0-5 test, with the test being significant only on the non-dominant side. Meanwhile, in the K-test, these goalkeepers performed better than full-backs, strikers, and wide midfielders, although not significantly [61]. A similar trend was observed for O19 goalkeepers. In both the 10-5 m sprint and the shuttle run (SR), goalkeepers performed worse than other positions, with the exception of centre-backs [17]. On the other hand, female O19 goalkeepers performed above average and outperformed other positions except for forwards in the pro-agility shuttle test. However, the opposite was true for the Arrowhead test, where they had the lowest scores [47]. In the same test, goalkeepers also performed below average compared to other positions, with the exception of full-backs and central midfielders [66]. Finally, in the 12.5 m agility test, goalkeepers performed better than average and outperformed players in other positions [69]. All this suggests that goalkeepers tend to perform worse in directional agility tests than other field positions. This may be due to a lower frequency and intensity of movement during competition [11]. Moreover, none established discriminatory thresholds (ROC/AUC, sensitivity/specificity, or cut-offs) by category or sex; observed differences are supported by means/effect sizes rather than diagnostic performance metrics.

### 4.3. Anaerobic Power

This area stands out as a future line of research, as anaerobic endurance has not been studied, especially in a specific sample of goalkeepers. It would be interesting to develop research based on category and sex, and to compare it with players in other positions.

### 4.4. Strength

Although the Wingate test is related to the assessment of anaerobic capacity, it is not alactic, making it more relevant to the assessment of anaerobic power (see Table 6). The results suggest a progressive development of anaerobic capacity in elite goalkeepers, confirming the importance of training anaerobic power from the lower categories. Future research should investigate whether mean relative power improves with age and category.

Absolute power in the force–velocity test improves with age and category. This trend is also observed for relative power, with the exception of the U19 category. This is interesting and warrants further investigation in future studies (see Table 7). When comparing the two tests by position, goalkeepers have higher absolute and peak power, but not relative power [66,70]. Therefore, the larger size and weight of goalkeepers may influence their performance.

Core strength and grip strength are related to the goalkeeper’s defensive performance. Both abilities are essential, as core strength allows for better stability and greater force generation in explosive actions, while grip strength allows for better ball control in blocks and clearances, providing greater safety in goalkeeping.

The results show an evolution in isometric core strength and its relationship with leg strength by age and category. This evolution is also observed for grip strength, with the exception of 31 U16 goalkeepers [45], who showed slightly lower values than the U14 group. Compared with other positions, goalkeepers show better performance in all tests [63].

When assessing isokinetic strength, it was noted that data were only recorded for the male category over 19 years of age, which prevented comparisons by age or sex. This highlights an area for future research based on the measurements in Table 8. The results suggest asymmetries between the legs, with the dominant or right leg showing greater strength. Two studies found no significant differences between legs in extension [74,75], suggesting the need to strengthen the weaker leg to prevent imbalances that could lead to injury and to optimise force production. Furthermore, the discrepancy between flexion and extension appears to be significant, except in the 24 goalkeepers from the Greek first division, where the dominant leg showed a more balanced relationship [73]. Previous studies have identified a 15% muscular asymmetry as a risk factor for injury [85]. This imbalance between flexors and extensors may increase the risk of injury in goalkeepers who rely on explosiveness to change direction and jump. Finally, when analysing movement speed (between 30°/s and 240°/s), we observed a constant decrease in torque in all samples. This suggests that goalkeepers should train in different speed ranges to improve their performance in explosive actions.

The scientific literature suggests that goalkeepers generate more force with the lower extremity muscles, perhaps due to their greater weight compared to other players [22]. This review is consistent with the literature on extension strength [53,72,74,75], but not on flexion, where values are comparable to certain positions such as centre-backs [72,74,75]. However, better performance has also been found for peak flexion strength, but not for relative strength [73].

During a match, goalkeepers perform between 3.8 ± 2.3 [86] and 15 ± 10 jumps per match [11]. Although this number may seem low, it represents almost half of their total interventions, with an average of 36 ± 4 interventions per match [10].

The most common type of jump is the countermovement jump (CMJ), which uses the stretch-shortening cycle (SSC) to improve vertical jump performance. When compared to the free arm CMJ (CMJ HF), the contribution of the arms is evident, increasing jump height by 22.6% in children and 18.7% in adults [87]. In general, males outperform females in this test [88].

Results suggest that CMJ height improves with age and level of competition, with a few exceptions such as 31 Greek U-16 and 11 U-19 goalkeepers who showed lower than expected performance [45]. In the CMJ HF, an improvement was observed from U-16 to U-19, but not in goalkeepers over the age of 19. In particular, Czech first division goalkeepers over 19 years of age increased their jump height by 5.01 cm when using their arms [44].

The squat jump (SJ), performed from a half-squat position without countermovement, shows comparable concentric strength in U-16 and U-19 goalkeepers, with the exception of 12 goalkeepers from the Tunisian first division who recorded lower values [68]. Overall, horizontal jumping performance (SJ) tends to improve with age, with the notable exception of four goalkeepers from the Brazilian Fourth Division who performed below average [62].

Few studies have examined horizontal jumping (SJ), despite its importance in assessing horizontal force production. Male goalkeepers generally improve with age, whereas female goalkeepers tend to perform at a level comparable to U-14 and U-16 field players.

Female goalkeepers show a high variability in CMJ height, ranging from 28.0 ± 3.0 cm to 37 ± 1.50 cm, making direct comparisons with male goalkeepers difficult. Notably, three US First Division goalkeepers performed remarkably well in this test [47], matched German U19s in CMJ HF [43], and outperformed Czech first division U19s [44].

Comparing the results with other positions, both male and female goalkeepers tend to be among the highest jumpers in the team, regardless of the test used [47,51,55,56,58]. However, in some samples, other positions—particularly centre-backs—perform better [17,48,49,57,65,66,69]. Nevertheless, goalkeepers may, in some cases, be among the lowest jumpers [63]. Therefore, goalkeepers require a high vertical jump capacity, which should be a key component of their training. However, this attribute should also be emphasised in the preparation of other positions, such as central defenders.

### 4.5. Flexibility

Flexibility and range of motion (ROM) are crucial for goalkeepers, allowing them to stretch and move efficiently to reach balls that would otherwise be out of reach with a more limited range of motion. Table 10 shows that flexibility scores in the sit-and-reach test remain consistent, with the exception of higher scores in Costa Rican goalkeepers [49]. Two studies reported an increase in flexibility with age from U14 to U16 [45,51], followed by a decrease in U19 goalkeepers, probably due to training-induced stiffness. It is possible that U19 goalkeepers need to adopt specific training strategies to prevent the decrease in flexibility observed in this category. However, an improvement was observed in goalkeepers over the age of 19, suggesting that structured flexibility training may be beneficial [63]. No female goalkeeper samples were found in flexibility or ROM tests, highlighting a potential research gap.

Goalkeepers showed greater ankle dorsiflexion and hip internal rotation in players from the Spanish Football Federation [52] compared to those from the Qatar Stars League [40] (see Table 11). In addition, passive hip flexion and extension were similar in both legs. These findings, together with the consistent flexibility across categories, suggest that goalkeepers require extensive ROM and can achieve higher values with structured training, as ROM varies between leagues and competition levels.

Consistent with the previous literature [22], goalkeepers demonstrate greater hip and leg extension ROM than outfield players [49,51,63]. However, this claim is contradicted in the U14 category, where goalkeepers rank second in flexibility, with central midfielders showing the highest values [51,63]. When analysing this claim on the basis of ROM, goalkeepers are often outperformed by players in other positions in the passive knee extension test [40] and straight leg raising test [66].

When comparing positions, goalkeepers have the highest hip ROM [53]. However, they show poorer results in internal rotation compared to other positions [40,52], but superior results in external rotation, hip flexion, and abduction [52]. In the Brent Knee Fall Out test, goalkeepers showed greater ROM than players in other positions [40]. For ankle flexion and extension, goalkeepers have greater mobility than outfield players [40,52].

### 4.6. Dynamic Balance and Coordination

Coordination, regulated by the central nervous system (CNS), is essential for goalkeepers and relies on motor skills and spatial awareness [44,89]. They maintain activation and balance in stance and synchronise movements with the opponent’s shot using the stretch-shortening cycle (SSC) to improve performance through muscle elasticity, energy reuse, and neural activation [85,90]. Inter- and intramuscular coordination partly regulate static and dynamic stability.

Table 12 shows that greater stability in the non-dominant leg of goalkeepers can be attributed to its role in passing and goal kicks. This suggests that experience and footwork training could improve the stability of the dominant leg. Additionally, goalkeepers excel in posteromedial and medial stability, but have less control in anterior, anterolateral, and lateral directions, which could affect their defensive performance. However, there is a significant lack of relevant studies, making this a potential avenue of research for youth categories and sex-based analysis.

When comparing stability performance by position, goalkeepers demonstrated the highest performance in all phases of the SEBT [1]. In the Y-balance test, goalkeepers ranked second in stability, with forwards showing the highest performance [64]. Furthermore, in the dominant leg, central midfielders showed greater stability, whereas in the non-dominant leg, goalkeepers showed superior performance [67].

The main limitation of this review lies in the scarcity of studies focused exclusively on goalkeepers, particularly female athletes and youth categories, as well as the use of small sample sizes, which undermines the reliability of the findings. Methodological heterogeneity across studies further complicates data comparison. Although PRISMA 2020 guidelines were followed and multiple databases in various languages were consulted, the limited availability of relevant literature required a certain degree of flexibility in the selection process. There is a clear need for research specifically targeting male and female goalkeepers across all age groups, using standardised tests that assess key abilities such as movement speed, reaction time, and neuromuscular performance. Moreover, future studies should explore critical aspects such as penalty kicks, given their significance in position-specific performance.

## 5. Conclusions

There are significant differences between goalkeepers and outfield players in terms of their physical demands. Agility should be considered one of the fundamental pillars of goalkeeper-specific training. Neuromuscular performance varies significantly depending on age, sex, and competitive level, particularly in skills such as linear speed, agility, change-of-direction speed, strength, and power. In contrast, flexibility does not show a clear progression with age, suggesting the need for specific strategies to maintain or improve it. In the case of female goalkeepers, there is a notable gap in the scientific literature regarding their performance based on sex and category, which limits the ability to design training programmes truly tailored to their specific needs.

## 6. Practical Applications and Recommendations

This review provides relevant insights for physical trainers and coaches by outlining the neuromuscular performance factors that need to be addressed to optimise goalkeeper performance. Based on these findings, agility should be prioritised in training programmes, with a particular focus on high-intensity, short-duration tasks.

The data presented can be used as a benchmark for physical performance in goalkeeper development and training within football, sports academies, and national associations. These findings may help to inform goalkeeper selection decisions or identify areas for improvement in neuromuscular performance, allowing for more targeted training plans.

In this review, we observed a number of tests that were not performed in other studies. Therefore, we recommend that the following tests be used to train and assess key skills in goalkeepers:

- Agility: Simple and complex Reactive Agility Tests (RAS)
- Speed with changes in direction: *t*-test, and for more specificity, the S-Keeper or LS-Keeper tests
- Linear speed: Linear sprint test up to 30 m, with measurements at 10 and 20 m. In particular, the 20 m sprint should be prioritised, as it is widely used to assess acceleration/sprint performance in soccer cohorts [91,92].
- Explosive strength (lower limbs): Countermovement jump (CMJ) test, as it is related to the stretch-shortening cycle (SSC). Additionally, we recommend assessing CMJ with free hands (CMJ HF) to better understand arm involvement in jumps. And the tests of isometric trunk and hand grip to evaluate the upper body.
- Flexibility: Sit-and-Reach test.
- Dynamic stability: Y-balance test

On the other hand, jump platforms are often used for jumping tests. Many studies also use athletic tracks and stopwatches to measure speed and agility. However, we recommend that these tests are carried out on the same surfaces that the athletes actually compete on, with goalkeepers wearing their specific footwear to better simulate match conditions. In addition, for greater accuracy, we recommend the use of photocells to measure speed, as they provide more accurate and reliable data compared to traditional stopwatch methods.

## Figures and Tables

**Table 1:** Methodological quality and risk of bias assessment in Analytical Cross-Sectional (JBI Critical Appraisal Checklist for Analytical Cross-Sectional Study; [36].

Study	1	2	3	4	5	6	7	8	RESULT
Knoop et al. [43]	+	+	+	+	-	-	+	+	INCLUDE
Zahálka et al. [44]	+	?	-	+	-	-	+	+	INCLUDE
Nikolaidis et al. [45]	+	+	+	+	+	-	?	+	INCLUDE
Herveou et al. [46]	+	+	+	+	-	-	+	-	INCLUDE

(1) Were the criteria for inclusion in the sample clearly defined? (2) Were the study subjects and the setting described in detail? (3) Was the exposure measured in a valid and reliable way? (4) Were objective, standard criteria used for measurement of the condition? (5) Were confounding factors identified? (6) Were strategies to deal with confounding factors stated? (7) Were the outcomes measured in a valid and reliable way? (8) Was appropriate statistical analysis used? Score: + = the content is appropriate; - = the content is not appropriate; ? = the content is unclear.

**Table 2:** Methodological quality and risk of bias assessment in Purely Cross-Sectional (JBI Critical Appraisal Checklist for Studies Reporting Prevalence Data; [37]).

Study	1	2	3	4	5	6	7	8	9	RESULT
Lockie et al. [47]	+	+	-	+	+	+	+	+	+	INCLUDE
Jiménez et al. [48]	+	+	-	-	+	+	+	+	+	INCLUDE
AlTaweel et al. [1]	+	+	-	+	+	+	+	+	+	INCLUDE
Serrano-Sanabria et al. [49]	+	+	-	+	+	-	+	+	+	INCLUDE
Rebelo et al. [50]	+	+	-	+	+	+	+	+	+	INCLUDE
Deprez et al. [51]	+	+	+	+	+	+	+	+	+	INCLUDE
Lopez-Valenciano et al. [52]	+	+	-	+	+	+	+	-	+	INCLUDE
AlTaweel et al. [53]	+	+	-	-	+	+	+	+	+	INCLUDE
Soyler and Kayantas [54]	+	+	-	+	+	-	-	+	+	INCLUDE
Sporis et al. [55]	+	+	-	+	+	?	+	+	+	INCLUDE
Pivovarniček et al. [42]	-	+	-	?	+	+	-	-	+	EXCLUDE
Kovačević et al. [56]	+	+	-	+	+	?	+	+	+	INCLUDE
Carpes et al. [57]	+	+	-	+	+	+	+	-	+	INCLUDE
Loureiro and Ferrari [58]	+	+	-	+	+	-	+	-	+	INCLUDE
Ravagnani et al. [59]	+	+	-	+	+	?	+	+	-	INCLUDE
Bizati [60]	+	+	-	+	+	+	+	+	+	INCLUDE
Bujnovky et al. [61]	+	+	-	-	+	+	+	+	+	INCLUDE
Boone et al. [17]	+	+	-	+	+	?	+	+	+	INCLUDE
Sousa and Rodrigues [62]	+	+	-	-	+	-	+	+	+	INCLUDE
Nikolaidis et al. [63]	+	+	+	+	+	+	+	+	+	INCLUDE
Ates [64]	+	+	-	+	+	+	+	+	+	INCLUDE
Perez-Contreras et al. [65]	+	+	-	+	+	?	+	+	+	INCLUDE
González-Vargas and Gallardo-Pérez [66]	+	+	-	+	+	+	+	+	+	INCLUDE
Mahmoudi et al. [67]	+	+	-	+	+	+	+	+	+	INCLUDE
Ben Hassen et al. [68]	+	+	-	?	+	+	+	+	+	INCLUDE
Vagle et al. [69]	?	+	-	+	+	+	+	+	+	INCLUDE
Baroni and Leal Junior [70]	+	+	-	+	-	+	+	?	+	INCLUDE
Jadczak et al. [71]	+	+	-	+	-	+	+	?	+	INCLUDE
Ruas et al. [72]	+	+	-	+	+	+	+	?	+	INCLUDE
Tsiokanos et al. [73]	+	+	-	+	-	+	+	?	+	INCLUDE
Charneco et al. [74]	+	+	-	+	+	+	+	?	+	INCLUDE
Maciel Germano et al. [75]	+	+	-	+	-	+	+	?	+	INCLUDE

(1) Was the sample appropriate to address the target population? (2) Were study participants sampled in an appropriate way? (3) Was the sample size adequate? (4) Were the study subjects and the settings described in detail? (5) Was the data analysis conducted with sufficient coverage of the identified sample? (6) Were valid methods used for the identification of the condition? (7) Was the condition measured in a standard, reliable way for all participants? (8) Was there appropriate statistical analysis? (9) Was the response rate adequate, and if not, was the low response rate managed appropriately? Score: + = the content is appropriate; - = the content is not appropriate; ? = the content is unclear.

**Table 3:** Results of the specific agility test for football goalkeepers (Mean ± SD); [43].

			RAS (Reaction and Action Speed) (s)					
Sex/Category	Age (Years)	*N*o. GK	Bottom Left	Bottom Right	Top Left	Top Right	Top Left- Bottom Right	Top Right- Bottom Left
MALE: U14	14.1 ± 0.3	13	1.40 ± 0.10	1.39 ± 0.12	1.59 ± 0.13	1.58 ± 0.12	5.20 ± 0.41	5.09 ± 0.59
MALE: U16		There is no specific agility test focused on this sample						
MALE: U19	18.4 ± 0.8	10 (1^st^gk)	1.25 ± 0.07	1.21 ± 0.06	1.38 ± 0.06	1.38 ± 0.04	4.28 ± 0.23	4.20 ± 0.20
	17.7 ± 0.7	11 (2^st^gk)	1.31 ± 0.05	1.24 ± 0.06	1.44 ± 0.08	1.41 ± 0.08	4.51 ± 0.24	4.43 ± 0.20
MALE and WOMEN: Over 19			There is no specific agility test focused on this sample					

U = under; *N*o. GK = is the number of goalkeepers; (1^st^ gk) = is the first goalkeeper, or the starting goalkeeper; (2^st^gk) = is the second goalkeeper, or the substitute goalkeeper; SD = Standard Deviation.

**Table 4:** Results of the sprint values for football goalkeepers (Mean ± SD).

Sex/Category/Selected Studies		Level	Surface	Testing Instruments	*N*o. GK	Sprint (s)			
						5 m	10 m	20 m	30 m
MALE U14: U14	Knoop et al. [43]	Elite lower categories in Germany	Artificial grass	Photocell gates (Sportronic GmbH, Schopfheim, Germany)	13	NS	1.98 ± 0.08	NS	NS
	Deprez et al. [51]	Elite lower categories in Belgium	Running track	Witty gate (Microgate Srl, Bolzano, Italy)	37	1.18 ± 0.09	NS	NS	4.96 ± 0.31
MALE: U16	Deprez et al. [51]	Elite lower categories in Belgium	Running track	Witty gate (Microgate Srl, Bolzano, Italy)	25	1.12 ± 0.08	NS	NS	4.57 ± 0.27
	Bujnovky et al. [61]	Top division of the Czech league	Artificial grass	Photocells Speed Trap II (Brower Timing System, Draper, UT, USA).	9	1.13 ± 0.07	1.9 ± 0.1	2.55 ± 0.11	NS
	Perez-Contreras et al. [65]	U15 Chilean national team	NS	Witty gate (Microgate Srl, Bolzano, Italy)	ND	NS	1.9 ± 0.1	NS	4.4 ± 0.1
MALE: U19	Knoop et al. [43]	Elite lower categories in Germany	Artificial grass	Photocell gates placed (Sportronic, Germany)	10	NS	1.83 ± 0.03	NS	NS
					11	NS	1.89 ± 0.05	NS	NS
	Serrano Sanabria et al. [49]	Elite lower categories in Costa Rica	NS	Newtest photocell (Newtest Oy, Oulu, Finland)	9	NS	1.89 ± 0.07	NS	NS
	Rebelo et al. [50]	Portugal U19 National League	Artificial grass	Photoelectric cells, Speed Trap II (Brower Timing Systems, Draper, UT, USA)	9	1.03 ± 0.06	NS	NS	4.31 ± 0.18
	Deprez et al. [51]	Elite lower categories in Belgium	Running track	Witty gate (Microgate Srl, Bolzano, Italy)	20	1.08 ± 0.05	NS	NS	NS
	Kovačević et al. [56]	Elite lower categories in Croatia	NS	Infrared photocells (NS)	7	NS	NS	NS	4.24 ± 0.11
	Perez-Contreras et al. [65]	U17 Chilean national team	NS	Photocells (Witty gate, Microgate Srl, Bolzano, Italy)	ND	NS	1.9 ± 0	NS	NS
	Ben Hassen et al. [68]	First Division in Tunisia	Artificial grass	Ipad 11 Pro (Apple Inc., Cupertino, CA, USA) (240 fps; 1080p; App “*My sprint*”)	12	NS	2.14 ± 0.08	3.55 ± 0.12	4.86 ± 0.18
MALE: OV	Soyler and Kayantas [54]	Second League in Ankara	NS	Photocell doors SMARTSPEED timing gates (Fusion Sport, Brisbane, QLD, Australia)	3	NS	1.48 ± 0.29	NS	NS
	Sporis et al. [55]	First National League Croatia	NS	Telematic photocell system (RS sport, Zagreb, Croatia)	30	1.45 ± 0.7	2.35 ± 0.8	3.51 ± 0.9	NS
	Ravagnani et al. [59]	Brazil First Division	Running track	Unmarked stopwatch (NS)	2	NS	NS	NS	4.3 ± 0.2
	Bizati [60]	Turkish Super League	Natural grass	Powertimer (Newtest Oy, Oulu, Finland)	3	0.94 ± 0.01	1.74 ± 0.05	2.94 ± 0.08	NS
	Boone et al. [17]	Belgian First Division	Field	Fotocell (Ergo Tester, Pisa, Italy)	17	1.46 ± 0.07	NS	NS	NS
WOMEN: OVER 19	Lockie et al. [47]	American Women’s First Division	NS	One timing gate (TC Timing System; Brower Timing, Draper, UT, USA)	3	1.188 ± 0.0	2.041 ± 0.0	NS	4.864 ± 0.0
	Loureiro and Ferrari [58]	Female players in Brazil	NS	Handheld chronometer (NS)	3	NS	NS	3.01± 0.03	NS
	González Vargas and Gallardo Pérez [66]	Primera división chilena	Natural grass	Chronojump Boscosystem photocells, version 1.7.0 (Chronojump, Barcelona, Spain)	3	NS	NS	NS	4.94 ± 0.3
	Vagle et al. [69]	Norwegian Premier League Female	Artificial grass	MuscleLab photocells (Ergotest Innovation AS, Porsgrunn, Norway).	14	NS	NS	3.21 ± 0.14	4.57 ± 0.20

U = under; *N*o. GK = is the number of goalkeepers; NS = Not specified; SD = Standard Deviation; s = seconds.

**Table 5:** Results of the *t*-test for football goalkeepers (Mean ± SD).

							*T* TEST (s)	
Sex/Category/Selected Studies		Level	Surface	Testing Instruments	*N*o. GK	*T* TEST	Left	Right
MALE: U14	Deprez et al. [51]	Elite categories in Belgium	NS	Witty gate (Microgate Srl, Bolzano, Italy)	37	NS	8.95 ± 0.34	8.99 ± 0.34
MALE: U16	Deprez et al. [51]	Elite categories in Belgium	NS	Witty gate (Microgate Srl, Bolzano, Italy)	25	NS	8.69 ± 0.32	8.66 ± 0.31
MALE: U19	Rebelo et al. [50]	First division in Portugal.	Artificial grass	Photoelectric cells, Speed Trap II (Brower Timing Systems, Draper, UT, USA)	9 elite	9.02 ± 0.33	NS	NS
	Deprez et al. [51]	Elite categories in Belgium	NS	Witty gate (Microgate Srl, Bolzano, Italy)	20	NS	8.52 ± 0.29	8.61 ± 0.32
MALE and WOMEN: Over 19			There is no *t*-test focused on this sample.					

U = under; *N*o. GK = is the number of goalkeepers; NS = Not specified; SD = Standard Deviation; s = seconds.

**Table 6:** Results of Wingate test for football goalkeepers (Mean ± SD).

Sex/Categories	Selected Studies	Level	*N*o. GK	Wingate (WAnT): Cycle Ergometer (Monark Exercise AB, Vansbro, Sweden)			
				PP (W)	AP (W)	RPP (W/kg)	RAP (W/kg)
MALE: U14	Nikolaidis et al. [63]	Elite categories in Greece	3	576.13 ± 89.19	444.77 ± 75.10	9.95 ± 0.88	7.68 ± 0.91
MALE: U16	Nikolaidis et al. [45]	Elite categories in Greece	31	629.9 ± 157.2	470.1 ± 121.4	NS	NS
	Baroni and Leal Junior [70]	National level of Brazil.	3	737.57 ± 59.77	580.17 ± 52.61	NS	8.21 ± 0.49
	Nikolaidis et al. [63]	Elite categories in Greece	8	772.55 ± 140.38	569.04 ± 104.16	10.47 ± 1.78	7.72 ± 1.40
MALE: U19	Nikolaidis et al. [45]	Elite categories in Greece	11	847.1 ± 122.8	612.6 ± 57.7	NS	NS
MALE: Over 19	Nikolaidis et al. [45]	Elite categories in Greece	24	904.0 ± 93.2	659.4 ± 66.6	NS	NS
	Nikolaidis et al. [63]	Elite categories in Greece	15	888.53 ± 108.09	656.68 ± 71.95	11.0 ± 0.62	8.16 ± 0.71
WOMEN: Over 19		There are no force velocity test values centred on this sample.					

U = under; *N*o. GK = is the number of goalkeepers; SD = Standard Deviation; NS = Not specified; PP = Peak power; AP = Average power; RPP = Relative peak power; RAP = Relative average power; W = watts; kg = kilogram.

**Table 7:** Results of strength and speed tests for football goalkeepers (Mean ± SD).

Sex/ Categories	Selected Studies	Level	Testing Instruments	*N*o. GK	Force Velocity		Hand Grip (kg)		IT (kg)	Trunk/Legs (kg)
					AP (W)	RP (W/kg)	Right	Left		
MALE: U14	Nikolaidis et al. [63]	Elite categories in Greece	Cycle ergometer (Monark Ergomedics, Sweden) and handgrip dynamometre (Takei, Japan)	3	645.89 ± 112.41	11.30 ± 2.48	35.37 ± 7.70	33.73 ± 6.34	74.67 ± 9.25	102.67 ±17.11
MALE: U16	Nikolaidis et al. [45]			31	702.0 ± 260.8	11.4 ± 3.2	33.2 ± 11.1	31.1 ± 10.3	88.3 ± 22.1	112.2 ± 24.9
	Nikolaidis et al. [63]			8	952.26 ± 133.84	13.12 ± 3.12	41.35 ± 9.00	38.06 ± 9.20	98.63 ± 17.38	120.38 ±23.21
MALE: U19	Nikolaidis et al. [45]			11	1190.6 ± 298.3	14.9 ± 3.7	45.6 ± 9.0	40.8 ± 7.2	122.7 ± 25.7	132.4 ± 36.7
MALE: Over 19	Nikolaidis et al. [45]			31	1165.8 ± 235.0	14.2 ± 2.8	51.9 ± 6.2	49.6 ± 5.5	148.5 ± 19.0	181.4 ± 27.2
	Nikolaidis et al. [63]			15	1135.71 ± 209.24	14.09 ± 2.3	50.75 ± 5.28	48.31 ± 6.47	146.09 ± 16.39	174 ± 26.56
WOMEN: Over 19		There are no force velocity test values centred on this sample.								

U = under; *N*o. GK = is the number of goalkeepers; NS = Not specified; W = watts; SD = Standard Deviation; W = watts; kg = kilogram; AP = Absolute power; RP = Relative power; IT = Isometric trunk.

**Table 8:** Results of isokinetic strength tests as a function of grades for football goalkeepers (Mean ± SD).

Selected Studies/Male Over19		*N*o. GK and Level	Laterality	Peak Torque (Nm); Isokinetic Dynamometer (Cybex 340, Rosemont, IL, USA)			
				(30°/s)	(60°/s)	(180°/s)	(240°/s)
Flexion	Tsiokanos et al. [73]	24GK First division in Greece	Dom	355 ± 45	312 ± 44	198 ± 30	NS
	Charneco Salguero et al. [74]	32GK Spanish first and second division	Right leg	NS	143.66 ± 20.30	119.33 ± 19.04	107.13 ± 19.93
			Left leg	NS	129.13 ± 27.71	106.23 ± 22.70	95.37 ± 20.02
	Maciel Germano et al. [75]	16GK Third and fourth division of Brazil	Dom	NS	160.59 ±17.23	NS	NS
			NDom	NS	154.63 ± 25.24	NS	NS
Extension	Charneco Salguero et al. [74]	32GK Spanish first and second division	Right leg	NS	245.47 ± 46.15	195.07 ± 23.56	162.47 ± 20.32
			Left leg	NS	246.25 ± 38.32	192.73 ± 27.88	160.60 ± 23.25
	Maciel Germano et al. [75]	16GK Third and fourth division of Brazil	Dom	NS	282.72 ± 51.35	NS	NS
			NDom	NS	283.36 ± 41.74	NS	NS

*N*o. GK = is the number of goalkeepers; SD = Standard Deviation; Dom = Dominant leg; NDom = Non-dominant leg; NS = Not specified; °/s = Degrees per second.

**Table 9:** Results jump tests for football goalkeepers (Mean ± SD).

Sex/Category/Selected Studies		Level	Testing Instruments (JUMP)	*N*o. GK	CMJ (cm)	SJ (cm)	CMJ HF (cm)	HJ (cm)
MALE U14	Deprez et al. [51]	Elite categories in Belgium	Optojump (Microgate Srl, Bolzano, Italy).	37	30.4 ± 5.8	NS	NS	200 ± 22
	Nikolaidis et al. [63]	Elite categories in Greece	Optojump (Microgate Srl, Bolzano, Italy).	3	26.71 ± 7.84	NS	NS	NS
MALE: U16	Knoop et al. [43]	Elite categories in Germany	Contact platform (Haynl-Elektronik GmbH, Schönebeck (Elbe), Germany).	13	NS	NS	36.0 ± 4.3	NS
	Nikolaidis et al. [45]	Elite categories in Greece	Optojump (Microgate Srl, Bolzano, Italy).	31	31.3 ± 8.9	NS	NS	NS
	Deprez et al. [51]	Elite categories in Belgium	Optojump (Microgate Srl, Bolzano, Italy).	25	35.5 ± 5.9	NS	NS	221 ± 20
	Nikolaidis et al. [63]	Elite categories in Greece	Optojump (Microgate Srl, Bolzano, Italy).	8	35.81 ± 7.52	NS	NS	NS
	Perez-Contreras et al. [65]	U15 Chilean national team	DmJump^®^ contact platform (DMJUMP, Santiago, Chile).	NS	39.3 ± 4.9	35.4 ± 4.8	NS	NS
MALE: U19	Knoop et al. [43]	Elite categories in Germany	Contact platform (Haynl-Elektronik GmbH, Schönebeck (Elbe), Germany).	10 (1^st^gk)	NS	NS	54.7 ± 5.8	NS
				11 (2^st^gk)	NS	NS	50.4 ± 4.2	NS
	Nikolaidis et al. [45]	Elite categories in Greece	Optojump (Microgate Srl, Bolzano, Italy).	11	32.8 ± 8.7	NS	NS	NS
	Serrano Sanabria et al. [49]	Elite lower categories in Costa Rica	Force platform “Newtest”(Newtest Oy, Oulu, Finland).	9	39 ± 5.59	31.4 ± 3.5	NS	NS
	Rebelo et al. [50]	First division U19 in Portugal	Special mat (Digitime 1000, NS, Finland)	9	41.9 ± 6.0	40.9 ± 5.0	NS	NS
	Deprez et al. [51]	Elite categories in Belgium	Optojump (Microgate Srl, Bolzano, Italy).	20	38.4 ± 4.4	NS	NS	230 ± 16
	Kovačević et al. [56]	Elite categories in Croatia	Quattro jump (Kistler Instrumente AG, Winterthur, Swizerland, 2008)	7	NS	NS	NS	272 ± 11
	Perez-Contreras et al. [65]	U17 Chilean national team	DmJump^®^ contact platform (DMJUMP, Santiago, Chile).	NS	37.8 ± 5.7	35.9 ± 2.9	NS	NS
	Ben Hassen et al. [68]	First division in Tunisia	My-Jump 2 app (v. 5.0.5) (Madrid, Spain)	12	NS	29.2 ± 5.0	NS	NS
MALE: Over 19	Zahálka et al. [44]	First division Czech	Kistler B8611A (Instrumente AG, Winterthur, Switzerland)	25	40.06 ± 3.48	36.1 ± 3.4	45.07 ± 3.22	NS
	Nikolaidis et al. [45]	Elite categories in Greece	Optojump (Microgate Srl, Bolzano, Italy).	31	37.7 ± 7.2	NS	NS	NS
	Herveou et al. [46]	French forth division	Optojump (Microgate Srl, Bolzano, Italy).	11	41.6 ± 5.5	38.5 ± 4.5	NS	NS
	Jiménez et al. [48]	Spanish Second Division B	kistler Quatro Jump (Force Platform) (Kistler Instrumente AG, Winterthur, Switzerland).	2	38.76 ± 1.67	36.9 ± 3.1	NS	NS
	Sporis et al. [55]	First National League Croatia	Quattro jump (Kistler Instrumente AG, Winterthur, Switzerland; 2008).	30	48.5 ± 1.5	46.8 ± 1.4	NS	NS
	Carpes et al. [57]	First division Brazil	Jumping platform (JUMP SYSTEM PRO, 1.0, NS, Chile)	9	50.0 ± 3.0	47.6 ± 4.5	NS	NS
	Bizati [60]	Turkish Super League	Powertimer (Newtest Oy, Oulu, Finland)	3	44.0 ± 5.07	41.2 ± 6.6	NS	NS
	Boone et al. [17]	Belgian first division	Jumping mat Ergo Tester (Globus Italia Srl, Codognè (TV), Italy).	17	45.6 ± 2.6	42.2 ± 2.9	NS	NS
	Nikolaidis et al. [63]	Elite categories in Greece	Optojump (Microgate Srl, Bolzano, Italy).	15	37.4 ± 6.87	NS	NS	NS
	Sousa and Rodrigues [62]	Forth division of Brazil	NS	4	37.2 ± 2.9	34.4 ± 5.0	NS	NS
WOMEN: Over 19	Lockie et al. [47]	American Women’s First Division	Force platform (Just Jump System) (Probotics, Huntsville, AL, USA).	3	NS	NS	54 ± 0.0	207 ± 0.0
	Loureiro and Ferrari [58]	Elite categories in Brazil	My Jump 2 app (v. 5.0.5) (Madrid, Spain)	3	37 ± 1.50	NS	NS	NS
	González Vargas and Gallardo Pérez [66]	Chilean First Division	Jumping platform (DMJump^®^2.0, NS, Chile)	3	28.0 ± 3.0	NS	NS	NS
	Vagle et al. [69]	Norwegian Premier League Female	Force platform (MuscleLab) (Ergotest Innovation AS, Stathelle, Norway).	14	32.6 ± 4.5	NS	NS	NS

U = under; *N*o. GK = is the number of goalkeepers; NS = Not specified; (1^st^gk) = is the first goalkeeper, or the starting goalkeeper; (2^st^gk) = is the second goalkeeper, or the substitute goalkeeper; SD = Standard Deviation; cm = centimetres; CMJ = Countermovement jump; SJ = Squat jump; CMJ HF = Countermovement jump with hands free; HJ = Horizontal jump.

**Table 10:** Results of flexibility tests for football goalkeepers (Mean ± SD).

Sex/Category/Selected Studies		Level	*N*o. GK	Sit and Reach (cm)	Modified Sit and Reach (cm)
MALE: U14	Deprez et al. [51]	Elite lower categories in Belgium	37	24.6 ± 6.1	NS
	Nikolaidis et al. [63]	Elite lower categories in Greece	3	19.75 ± 6.63	NS
MALE: U16	Nikolaidis et al. [45]	Elite lower categories in Greece	31	NS	19.4 ± 6.5
	Deprez et al. [51]	Elite lower categories in Belgium	25	29.1 ± 8.9	NS
	Nikolaidis et al. [63]	Elite lower categories in Greece	8	23.81 ± 5.90	NS
MALE: U19	Nikolaidis et al. [45]	Elite categories in Greece	11	NS	26.9 ± 6.8
	Serrano Sanabria et al. [49]	Elite categories in Costa Rican	9	43.11 ± 9.63	NS
	Deprez et al. [51]	Elite categories in Belgium	20	27.4 ± 4.3	NS
MALE: Over 19	Nikolaidis et al. [45]	Elite categories in Greece	31	NS	24.1 ± 6.8
	Carpes et al. [57]	First division Brazil	9	22.26 ± 4.14	NS
	Nikolaidis et al. [63]	Elite categories in Greece	15	25.65 ± 7.61	NS
WOMEN: Over 19		There are no sit-and-reach values centred on this sample.			

U = under; *N*o. GK = is the number of goalkeepers; NS = Not specified; SD = Standard Deviation; cm = centimetres.

**Table 11:** Results of range of movement tests for football goalkeepers (Mean ± SD).

MALE: OVER 19		*N*o. GK	Test	Dominant Leg	Non-Dominant Leg
López Valenciano et al. [52]	Spanish Football Federation	14	Passive hip flexion with knee flexed	150.9 ± 9.4°	151.8 ± 7.2°
			Passive hip flexion with knee extended	80.3 ± 10.1°	79.5 ± 10.7°
			Passive hip abduction	67.9 ± 7.6°	66.6 ± 9.8°
			Passive hip internal rotation	49.4 ± 10.5°	47.9 ± 6.3°
			Passive hip external rotation	50.8 ± 7.6°	48.5 ± 8.3°
			Ankle dorsiflexion with knee extended	36.6 ± 5.1°	37.0 ± 5.1°
			Passive hip extension	12.2 ± 7.4°	12.7 ± 7.8°
			Passive knee flexion	131.7 ± 10.9°	131.4 ± 13.2°
Wik et al. [40]	Qatar Stars League	19	Bent knee fall out	13.2 ± 4.7°	13.0 ± 3.7°
			Passive knee extension	87.9 ± 11.7°	86.4 ± 10.8°
			Hip internal Rotation	33.5 ± 5.8°	34.2 ± 6.3°
			Ankle dorsiflexion with knee extended	11.1 ± 3.7°	10.9 ± 3.2°
González Vargas and Gallardo Perez [66]	Chilean First Division	3	The straight leg raises	107 ± 15.0°	106 ± 7.5°
AlTaweel et al. [53]	Saudi Arabian First Division	24	Hip ROM	19.79 ± 1.82°	

*N*o. GK = is the number of goalkeepers; ° = Degrees; SD = Standard Deviation.

**Table 12:** Table of values for dynamic balance tests (Mean ± SD).

Sex/Category/Selected Studies		*N*o. GK		Test	Phase of the Test	Dominant Leg (cm)	Non-Dominant Leg (cm)
MALE: U19	Mahmoudi et al. [67]	10	Iranian Professional League	Y-balance	Anterior	88.6 ± 7.2	94.1 ± 9.5
					Posterolateral	92.6 ± 8.3	95.6 ± 9.2
					Posteromedial	99.7 ± 8.3	103.8 ± 5.5
MALE: OVER 19	Ateş [64]	3	Turkish Second League	Y-balance	Anterior	69.6 ± 0.0	69.7 ± 0.0
					Posterolateral	118.2 ± 0.0	118.2 ± 0.0
					Posteromedial	117.7 ± 0.0	113.6 ± 0.0
MALE: OVER 19	AlTaweel et al. [1]	21	Saudi League	SEBT (Star Excursion Balance Test)	Anterior	48.23 ± 5.42	
					Anteromedial	50.95 ± 5.31	
					Medial	51.44 ± 5.66	
					Posteromedial	51.18 ± 5.89	
					Posterior	49.88 ± 6.79	
					Posterolateral	47.76 ± 5.92	
					Lateral	42.69 ± 5.72	
					Anterolateral	45.59 ± 5.0	

U = under; *N*o. GK = is the number of goalkeepers; SD = Standard Deviation; cm = centimetres.

## Data Availability

No new data were created or analysed in this study. All data supporting the findings are derived from previously published studies cited in the References. Data sharing is not applicable to this article.

## Abbreviations

The following abbreviations are used in this manuscript:

U	Under
*N*o. GK	Number Of Goalkeepers
1^st^gk	First Goalkeeper, Or The Starting Goalkeeper
2^st^gk	Second Goalkeeper, Or The Substitute Goalkeeper
SD	Standard Deviation
NS	Not Specified
s	Seconds
°	Degrees
°/s	Degrees Per Second
cm	Centimetres
W	Watts
kg	Kilogram
Dom	Dominant Leg
NDom	Non-Dominant Leg
AP	Average Power
RP	Relative Power
PP	Peak Power
RPP	Relative Peak Power
RAP	Relative Average Power
IT	Isometric Trunk
CMJ	Countermovement Jump
SJ	Squat Jump
CMJ HF	Countermovement Jump With Hands Free
HJ	Horizontal Jump

## Appendix A

**Table A1:** Effects reported by the selected studies on the neuromuscular performance of goalkeepers.

Name of Authors and Study Type	Objective Focus	Sample and Groups	Measurement and Test	Main Outcomes
Knoop et al. [43] *Analytical Cross-Sectional*	Develop and evaluate a new agility test for goalkeepers between starting and substitutes GK and categories U14/U19	GK (*n* = 34 Male): (*n* = 10) U19 Starting Gk (18.4 ± 0.8 y) (*n* = 11) U19 Backup GK (17.7 ± 0.7 y)/(*n* = 13) U14 (14.1 ± 0.3 y)	Linear Speed (Sprints (S10 m(m) in seconds (s)); Explosive Strength (CMJ (cm)); Agility (Reaction and Action Speed (RAS) Test (Single and Complex Test))	S10: (U14: 1.98 ± 0.08 s; U19 = 1.86 ± 0.05 s); CMJ (cm) (U14: 36.0 ± 4.3 cm; U19 = 47.8 ± 5.5 cm); Reaction and Action Speed (RAS) Test—Single Test: Bottom left (s) (U14: 1.40 ± 0.10 s; U19 = 1.28 ± 0.06 s) Bottom right (s) (U14: 1.39 ± 0.12 s; U19 = 1.23 ± 0.06 s)—RAS Complex Test: Top left (s) (U14: 1.59 ± 0.13 s; U19 = 1.41 ± 0.08 s); Top right (s) (U14: 1.58 ± 0.12 s; U19 = 1.39 ± 0.06 s); Top left—bottom right (s) (U14: 5.20 ± 0.41 s; U19= 4.40 ± 0.26 s); Top right—bottom left (s) (U14: 5.09 ± 0.59 s; U19 = 4.32 ± 0.23 s). All of the values present significant different *p* < 0.01)
Zahálka et al. [44] *Analytical Cross-Sectional*	Assessment of power strength and comparison of lower limb strength asymmetries	GK: (*n* = 25) O19 (26.5 ± 9.1 y)/Male	Explosive Strength (CMJ with arm, CMJ, SJ); Force asymmetry (ΔFmax)	CMJ with arm: 45.07 ± 3.22 cm; CMJ: 40.06 ± 3.48 cm; SJ: 36.09 ± 3.42 cm; ΔFmax: CMJ with arm: 8.61 ± 5.33%; CMJ: 7.06 ± 5.55%; SJ: 3.95 ± 3.48%. Fmax (N) CMJ with arm: 2210.0 ± 307.39; CMJ: 2173.48 ± 187.49 cm; SJ: 1815.24 ± 121.01; Frel (N) CMJ with arm: 2.56 ± 0.22 cm; CMJ: 2.51 ± 0.1; SJ: 2.10 ± 0.14
Nikolaidis et al. [45] *Analytical Cross-Sectional*	Assessment physical characteristics and physiological attributes and comparison between groups U16 years, U19, and over 19	Three age GK groups—Male/U16 U19, and O19	Strength (Wingate (WanT) (W) (Peak power (PP) and Mean power (MP)), Force–velocity test (Absolute and Relative power (AP, RP)), hand grip (kg), isometric trunk (T) and isometric trunk–legs (T/L) (kg)); Explosive Strength (Vertical jump (cm)); Flexibility (Modified sit and reach (MSR)(cm))	WAnT: PP (W) (U16 = 629.9 ± 157.2; U19 = 847.1 ± 122.8; O19 = 904 ± 93.2) MP (W) (U16 = 470.1 ± 121.4; U19 = 612.6 ± 57.7; O19 = 659.4 ± 66.6) (*p* < 0.001). Force–velocity test AP(W) (U16 = 702 ± 260.8; U19 = 1190.6 ± 298.3; O19 = 1165.8 ± 235) (*p* < 0.001). RP (W·kg^−1^) (U16 = 11.4 ± 3.2; U19 = 14.9 ± 3.7; O19 = 14.2 ± 2.8). VJ (cm) (U16 = 31.3 ± 8.9; U19 = 32.8 ± 8.7; O19 = 37.7 ± 7.2). Hand grip (kg) R (U16 = 33.2 ± 11.1; U19 = 45.6 ± 9.0; O19 = 51.9 ± 6.2) (*p* < 0.001) L (U16 = 31.1 ± 10.3; U19 = 40.8 ± 7.2; O19 = 49.6 ± 5.5) (*p* < 0.001). T (kg) (U16 = 88.3 ± 22.1; U19 = 122.7 ± 25.7; O19 = 148.5 ± 19) (*p* < 0.001). T/L (kg) (U16 = 112.2 ± 24.9; U19 = 132.4 ± 36.7; O19 = 181.4 ± 27.2) (*p* < 0.001). MSR (cm) (U16 = 19.4 ± 6.5; U19 = 26.9 ± 6.8; O19 = 24.1 ± 6.8) (*p* = 0.003)
Herveou et al. [46] *Analytical Cross-Sectional*	Evaluation of mechanical muscle capacities and strength–velocity profiles and to determine specific muscle qualities of football goalkeepers to optimise training programmes	GK: (*n* = 11) 24.3 ± 2.6 y/Male	Explosive Strength (SJ, CMJ (cm)); Strength (Upper and Lower limb stiffness, Upper and Lower limb force–velocity profile, Slope, Maximal theoretical force and velocity (N·m^−1^·kg^−1^))	Squat Jump (SJ): 38.5 ± 4.5 cm countermovement jump (CMJ): 41.6 ± 5.5 cm Lower limb stiffness: 304.2 ± 55.1 N·m^−1^·kg^−1^ Lower limb force–velocity profile:—Maximal theoretical force (F0): 34.3 ± 5.9 N·kg^−1^—Maximal theoretical velocity (V0): 3.2 ± 0.6 m·s^−1^—Slope (SF-V): −11.5 ± 4.0 N·s·m^−1^·kg^−1^) Upper limb force–velocity profile: Maximal theoretical force (F0): 13.6 ± 4.3 N·kg^−1^—Maximal theoretical velocity (V0): 3.7 ± 0.6 m·s^−1^—Slope (SF-V): −3.7 ± 1.1 N·s·m^−1^·kg^−1^
Lockie et al. [47] *Purely Cross-Sectional*	Document the existence of differences in anthropometry, power, linear speed, change in direction (COD), and specific resistance depending on the different playing positions	GK: (*n* = 3) O19 (20.5 ± 0 y)/Female	Explosive Strength (VJ (m), SH(m)); Linear Speed (5-10-30 m (s)); Speed with change in directions (COD) (Pro-agility shuttle (s), Arrowhead left and right (s))	Vertical jump (m) = 0.54 m; Standing Broad Jump(m) = 2.07 m; S5 (s) = 1.188 s; S10 (s) = 2.041 s; S30 (s) = 4.864 s; Pro-agility shuttle (s) = 4.935 s; Arrowhead left (s) = 9.199 right (s) = 9.201 s
Jiménez et al. [48] *Purely Cross-Sectional*	Evaluate and compare jumping power between players of different playing positions	GK: (*n* = 2) O19 (22.5 ± 2.12 y)/Male	Explosive Strength (SJ, CMJ); Strength (Maximal Force (Fmax) (%BW), maximal velocity (Vmax), and power (P) (W/Kg))	SJ (36.94 ± 3.14 cm) CMJ (38.76 ± 1.67 cm). Fmax (%BW): SJ (2.40 ± 0.09) CMJ (2.53 ± 0.13). Vmax (m s^−1^): SJ (2.79 ± 0.11) CMJ (2.91 ± 0.06). P (W/Kg): SJ (53.79 ± 3.12) CMJ (53.51 ± 4.31)
AlTaweel et al. [1] *Purely Cross-Sectional*	Know the differences in muscle strength and hip flexibility depending on the playing position	GK: (*n* = 24) O19 (23.58 ± 3.69 y)/Male	Strength (isokinetic strength: 90° flexion and extension, 180° flexion and extension, 90° Abductor and Adductor, 180° Abductor and Adductor); Mobility (HIP range of movement (ROM))	Isokinetic strength: 90° Flexion (100.68 ± 20.39) 90° Extension (155.13 ± 45.71): 180° Flexion (88.92 ± 20.09) 180° Extension (133.54 ± 47.13): 90° Abductor (63.73 ± 18.91) 90° Adductor (92.20 ± 46.41): 180° Abductor (41.78 ± 21.56) 180° Adductor (81.13 ± 44.85): HIP ROM (19.79 ± 1.82)
Serrano Sanabria et al. [49] *Purely Cross-Sectional*	To compare the anthropometry and neuromuscular capacities of football players based on their playing positions	GK: (*n* = 9) U19 (17.8 ± 1.2 y)/Male	Flexibility (Sit and Reach (SR)); Explosive Strength (CMJ, SJ (cm)); anaerobic endurance (Fatigue Index (FI)); Linear Speed (Sprint 10 m and 25 m (m/s))	SR (43.11 ± 9.63 cm); CMJ (39 ± 5.59 cm); SJ (31.44 ± 3.53 cm); FI (0.77 a ± 0.04); Vel. 10 m (m/s) (5.30 ± 0.37); Vel. 25 m (m/s) (6.47 ± 0.41)
Rebelo et al. [50] *Purely Cross-Sectional*	Know the anthropometry, physical aptitude and technical performance according to your playing position	GK (*n* = 18 Male): U19 elite (*n* = 9) (18.2 ± 0.6 y) and U19 non-elite (*n* = 9) (17.9 ± 0.4 y)	Velocity (5 m, 30 m (s)); Explosive Strength (SJ, CMJ); Strength (Peak Torque Extension (PT ex), Peak Torque Flexion PT flex (N m)); Agility (s)	SJ (Elite 40.9 ± 5.0 cm and Non-Elite 34.2 ± 6.0 cm), CMJ (Elite 41.9 ± 6.0 cm and Non-Elite 32.8 ± 1.4 cm), PT ext (N · m) (Elite 236 ± 33 and Non-Elite 202 ± 44) PT fl ex (N · m) (Elite 117 ± 35 and Non-Elite 91 ± 28) T 5 m (s) (Elite 1.03 ± 0.06 and Non-Elite 1.15 ± 0.16) T 30 m (s) (Elite 4.31 ± 0.18 and Non-Elite 4.56 ± 0.37) agility (s) (Elite 9.02 ± 0.33 and Non-Elite 9.39 ± 0.46) Yo-Yo IE2 (m) (Elite 992 ± 214 and Non-Elite 647 ± 247)
Deprez et al. [51] *Purely Cross-Sectional*	To know the differences in anthropometric and functional characteristics depending on their category and playing position	GK: (n = 82; Male) U15 (n = 37) (13.7± 0.6 y), U17 (*n* = 25) (15.8 ± 0.7 y), U19 (*n*= 20) (17.7± 0.6 y)	Flexibility (Sit-and-Reach (SR) (cm)); Linear Speed (Sprint S5 m, S30 m); Agility (*T*-Test (s))	SR (U15 (29.2 ± 5.7 cm) U17 (30.4 ± 6.0 cm) U19 (31.1 ± 6.2 cm)). S5 m: (U15 (1.10 ± 0.04 s) U17 (1.07 ± 0.03 s) U19 (1.05 ± 0.03 s)). S30 m: (U15 (4.51 ± 0.16 s) U17 (4.38 ± 0.14 s) U19 (4.29 ± 0.12 s)). T-Test: (U15 (9.87 ± 0.39 s) U17 (9.65 ± 0.35 s) U19 (9.48 ± 0.32 s))
Lopez-Valenciano et al. [52] *Purely Cross-Sectional*	To understand the range of motion (ROM) of the lower limbs in professional football players and to analyse the differences in ROM between goalkeepers and field players	GK: (*n* = 14) O19 (25.5 ± 5.0 y)/Male	Flexibility (Hip flexion with knee flexed (PHF KF); Hip flexion with knee extended (PHF KE); Hip extension (PHE); Hip abduction (PHA); Hip internal rotation (PHIR); Hip external rotation (PHER); Knee flexion (PKF); Ankle dorsiflexion with knee flexed (ADF KF); Ankle dorsiflexion with knee extended (ADF KE))	PHF KF: (Dom = 150.9 ± 9.4 y NDom = 151.8 ± 7.2); PHF KE: (Dom = 80.3 ± 10.1 y NDom = 79.5 ± 10.7); PHE: (Dom = 12.2 ± 7.4 y NDom = 12.7 ± 7.8); PHA: (Dom = 67.9 ± 7.6 y NDom = 66.6 ± 9.8); PHIR:(Dom = 49.4 ± 10.5 y NDom = 47.9 ± 6.3); PHER: (Dom = 50.8 ± 7.6 y NDom = 48.5 ± 8.3); PKF: (Dom = 131.7 ± 10.9 y NDom = 131.4 ± 13.2); AADF KF: (Dom = 37.5 ± 7.1 y NDom = 40.6 ± 4.7); ADF KE: (Dom = 36.6 ± 5.1 y NDom = 37.0 ± 5.1)
AlTaweel et al. [53] *Purely Cross-Sectional*	Know the differences in anaerobic power, dynamic stability, lower extremity strength and power depending on your playing position	GK: (*n* = 24) O19 (23.58 ± 3.69 y)/Male	Explosive Strength (Single Leg Vertical Jump (SLVJ)); Dynamic Stability (Star excursion balance test (SEBT))	SLVJ (16.32 ± 2.29); SEBT (Stability)—Anterior (48.23 ± 5.42); Anteromedial (50.95 ± 5.31); Medial (51.44 ± 5.66); Posteromedial (51.18 ± 5.89); Posterior (49.88 ± 6.79); Posterolateral (47.76 ± 5.92)
Kovačević et al. [56] *Purely Cross-Sectional*	Determine the differences in physical and physiological characteristics depending on their playing position	GK: (*n* = 7) U19 (17.06 ± 0.74 y)/Male	Explosive Strength (Standing Long Jump (SLJ); Vertical jump (VJ)); Linear Speed (Sprint 30 m (S30), in 60 m (S60), 5 × 10 m sprint (S5 × 10 m))	SLJ (2.72 ± 0.11); VJ (0.6 ± 0.07 m); S30 (4.24 ± 0.11 s); S60 (7.65 ± 0.19 s); S5 × 10 m: 11.04 ± 0.70 s. Significant differences in Standing Long Jump (SLJ) test between playing positions (*p* = 0.00). Post hoc tests showed differences between goalkeepers and other positions (*p* = 0.01)
Soyler and Kayantas [54] *Purely Cross-Sectional*	Evaluate the physical and physiological profiles, depending on their playing position	GK: (*n* = 3) O19 (26.51 ± 2.50 y)/Male	Flexibility (Sit–reach (SR) (cm)); Vertical jump (41.83 ± 2.72); Linear Speed (Sprint 10 m)	SR (22.26 ± 4.14 cm); VJ (41.83 ± 2.72); S10 (1.48 ± 0.29)
Sporis et al. [55] *Purely Cross-Sectional*	Determine the conditioning profile of youth players who play in different playing positions	GK: (*n* = 30) O19 (31.5 ± 2.3)/Male	Linear Speed (Sprint 5, 10 m, 20 m); Explosive Strength (SJ, CMJ)	S5 m: (1.45 ± 0.7); S10 m: (2.35 ± 0.8); S20 m: (3.51 ± 0.9); SJ: (46.8 ± 1.4); CMJ: (48.5 ± 1.5)
Carpes et al. [57] *Purely Cross-Sectional*	To assess and compare the fitness levels of football players who play different positions in the game	GK: (*n* = 9) O19 (27.1 ± 4.5 y)/Male	Anaerobic Power (RAST test (Pmáx (w/kg); Índice de Fatiga(w/seg)); Explosive Strength (SJ, CMJ (cm))	Pmáx (10.6 ± 0.6 w/kg); Índice de Fadiga (10.5 ± 2.2 w/seg); SJ (47.6 ± 4.5 cm); CMJ (50 ± 3 cm)
Loureiro and Ferrari [58] *Purely Cross-Sectional*	Analyse anthropometry and physical fitness based on your playing position	GK: (*n* = 3) O19 (25 ± 5.50 y)/Male	Linear Speed (Sprint 20 m (S20) (s)); Explosive Strength (CMJ (cm))	S20 (3.01 ± 0.03 s); CMJ (37 ± 1.50 cm)
Ravagnani et al. [59] *Purely Cross-Sectional*	Compare anthropometrics and physical performance based on your playing position	GK: (*n* = 2) O19 (24.0 ± 1.0 y)/Male	Linear Speed: (Sprint 30 m); Anaerobic Power (Rast test PTM (w/kg))	S30: (4.3 ± 0.2 s) (Excelente); Rast test PTM (w/kg) (7.4 ± 0.3))
Bizati [60] Purely *Cross-Sectional*	Evaluate and compare the physical and physiological characteristics of football players based on their playing positions	GK: (*n* = 3) O19 (22.67 ± 2.52 y)/Male	Linear Speed (Sprint 5 m, 10 m, 20 m); Explosive Strength (SJ, CMJ)	S5: (0.94 ± 0.01); S10: (1.74 ± 0.05); S20: (2.94 ± 0.08); SJ: (41.23 ± 6.64); CMJ: (44.00 ± 5.07)
Bujnovky et al. [61] *Purely Cross-Sectional*	Evaluate differences in speed, agility, aerobic, and anaerobic capacities depending on their playing position	GK: (*n* = 9) U16 (15.7 ± 0.5 y)/Male	Linear Speed (Sprint 5 m, 10 m, 20 m (s); Agility (agility 505 dominant (A505Dom) and non-dominant (A505ND), K-test (s))	S5 m (S5) (1.13 ± 0.07 s); S10 m (S10) (1.90 ± 0.10 s); S20 m (2.55 ± 0.11 s); A505Dom (2.59 ± 0.11 s); A505ND (2.61 ± 0.23 s); K-test (10.85 ± 0.40 s)
Boone et al. [17] *Purely Cross-Sectional*	Knowing the physical and physiological profile according to your playing position	GK: (*n* = 17) O19 y/Male	Linear Speed (S5 m); Agility (10–5 m; shuttle run (SR)); Explosive Strength (SJ, CMJ (cm))	5 m (1.46 ± 0.07 ^†,‡^ s); 10–5 m (0.76 ± 0.06 ^†,‡^ s); SR (12.32 ± 0.44 ^†,‡^ s); SJ (42.2 ± 2.9 *^,†,§^ cm); CMJ (45.6 ± 2.6 ^†^ cm)
Sousa and Rodrigues [62] *Purely Cross-Sectional*	Analyse the differences in vertical jump based on your playing position	GK: (*n* = 4) O19 (19.2 ± 2 y)/Male	Explosive Strength (SJ, CMJ (cm))	SJ (34.4 ± 5.0 cm); CMJ (37.2 ± 2.9 cm)
Nikolaidis et al. [63] *Purely Cross-Sectional*	Know the physical and physiological characteristics based on your playing position	GK: (*n* = 26 Male); (*n* = 3) U14 (13.23 ± 0.52 y); (*n* = 8) U17 (15.47 ± 0.83 y); (*n* = 15) O19 (20.45 ± 3.48 y)	Strength (Wingate Anaerobic Test (WanT) (Peak power (PP)(W), Relative peak power (RPP)(W·kg^−1^), Mean power (MP)(W), Relative mean power (RMP)(W·kg1)), Force–velocity test (AP (W), RP (W·kg^−1^)), Isometric strength ((RHG) Right-hand grip and Left (RLG) (kg), (T) Trunk (74.67 ± 9.25 kg) (T/L) Trunk/Legs (102.67 ± 17.11 kg)); Explosive Strength (Vertical jump (VJ) (cm); Flexibility (Sit and reach (SR) (cm))	U14: WanT (PP (576.13 ± 89.19 W), RPP (9.95 ± 0.88 W·kg^−1^) (MP) MP (444.77 ± 75.10 W); RMP (7.68 ± 0.91 W·kg^−1^)) Force–velocity test (AP (645.89 ± 112.41 W), RP (11.30 ± 2.48 W·kg^−1^)) VJ (26.71 ± 7.84 cm). Isometric strength (RHG (33.73 ± 6.34 kg), RLG (33.73 ± 6.34 kg), T (74.67 ± 9.25 kg), T/L (102.67 ± 17.11 kg)); Flexibility: SR (19.75 ± 6.63 cm). U17: WanT (PP (772.55 ± 140.38 W) RPP (10.47 ± 1.78 W·kg^−1^) MP (569.04 ± 104.16 W) RMP (7.72 ± 1.40 W·kg^−1^)) Force–velocity test (AP (952.26 ± 133.84 W), RP (13.12 ± 3.12 W·kg^−1^)) VJ (35.81 ± 7.52 cm). Isometric strength (RHG (41.35 ± 9.004 kg) LHG (38.06 ± 9.20 kg) T (98.63 ± 17.38 kg) T/L (120.38 ± 23.21 kg)) Flexibility: SR (23.81 ± 5.90 cm). Over19: WanT (PP (888.53 ± 108.09 W) RPP (11.00 ± 0.62 W·kg^−1^) MP (656.68 ± 71.95 W) RMP (8.16 ± 0.71 ** W·kg^−1^)) Force–velocity test (AP (1135.71 ± 209.24 W), RP (14.09 ± 2.30 W·kg^−1^)) VJ (37.40 ± 6.87 cm). Isometric strength (RHG (50.75 ± 5.28 kg) LHG (48.31 ± 6.47 kg) T (146.09 ± 16.39 kg) T/L (174.00 ± 26.56 kg)) Flexibility: SR (25.65 ± 7.61 cm)
Ates [64] Purely *Cross-Sectional*	Compare dynamic stability performance based on their playing positions	GK: (*n* = 3) O19 (23.3 Y)/Male	Dynamic Stability (Y-balance dominant and not dominant ((%, Reach Asymmetry (RA), and Composite (%)) (Anterior, Posteromedial, and Posterolateral))	Anterior (%) (Dom (69.6 ± 0); Ndom (69.7 ± 0); RA (0.11 ± 0)); Posteromedial (%) (Dom (118.2 ± 0); Ndom (118.2 ± 0); RA (0.001 ± 0)); Posterolateral (%) (Dom (117.7 ± 0); Ndom (113.6 ± 0); RA (4.09 ± 0)); Composite (%) (Dom (101.8 ± 0); Ndom (100.5 ± 0); RA (1.32 ± 0))
Perez-Contreras et al. [65] *Purely Cross-Sectional*	Understand the relationships between body composition and physical performance, depending on playing positions	GK: (*n* = Not defined, Male) U15 (15.2 ± 0.5 y) U17 (17.0 ± 0.5 y)	Linear Speed (Sprint 10, and 30 m) (s)); Explosive Strength (SJ, CMJ (cm))	U15: (S10 (1.9 ± 0.1 s); S30 (4.4 ± 0.1 s); SJ (35.4 ± 4.8 cm); CMJ (39.3 ± 4.8 cm)) U17: (S10 (1.9 ± 0 s); S30 (4.4 ± 0.1 s); SJ (35.9 ± 2.9 cm); CMJ (37.8 ± 5.7 cm))
González Vargas and Gallardo Pérez [66] Purely *Cross-Sectional*	Evaluate speed, strength, endurance, and flexibility based on their playing position	GK: (*n* = 3) O19 (25.3 ± 5.5 y)/Female	Linear Speed (Sprint 30 metros (S30) (s)); Agility (Arrowhead Agility Test (AAT)); Explosive Strength (CMJ (cm)); Flexibility (Deficit Balance (DB) (°), Dominant straight leg raise (DSR-R), and non-dominant (NDSR-R) (°))	S30(4.94 ± 0.3 s); AAT (9.18 ± 0.9 s); CMJ (28.0 ± 3.0 cm); DSR-R (107 ± 15.0°); NDSR-R (106 ± 7.6°) DB ≥ 8° (2/3)
Mahmoudi et al. [67] *Purely Cross-Sectional*	To assess the differences in static and dynamic stability between different positions of football players	GK: (*n* = 10) U19 (18.6 ± 1.1 s)/Male	Dynamic Stability (Y-Balance Test (YBT); Static Stability; Path length (PL (mm) (dominant leg, Eyes Open (DL EO), and non-dominant leg (NDL.EO); dominant leg, Eyes Closed (DL. EC) and non-dominant (NDL.EC); Double Leg Foam Surface Eyes Open (Dul. FS. EO), Double Leg Foam Surface Eyes Close (Dul. FS.EC))	Path length (PL (mm) (DL EO (1046.4 ± 152.1); (NDL.EO (1121.7 ± 171.7); DL. EC (2148 ± 507.1); Dul. FS. EO (1231.4 ± 192.8); Dul. FS.EC (2427.2 ± 503.1)). MV (mm/s) (DL EO (41.8 ± 6.1); NDL.EO (44.9 ± 6.8); DL. EC (73.7 ± 13.7); NDL. EC (85.94 ± 20.3); Dul. FS. EO (22.4 ± 3.5); ul. FS.EC (44.1 ± 9.1). AP (mm) (DL EO (8.0 ± 1.8); NDL.EO (8.9 ± 2.1); DL. EC (12.0 ± 1.7); NDL. EC (13.5 ± 3.4); Dul. FS. EO (9.4 ± 2.3); ul. FS.EC (11.8 ± 2.3). ML (mm) (DL EO (5.5 ± 0.9); NDL.EO (5.7 ± 0.5); DL. EC (11.9 ± 5.2; NDL. EC (11.3 ± 1.9); Dul. FS. EO (6.6 ± 0.7); ul. FS.EC (11.9 ± 2.8). Area(mm^2^) ML (mm) (DL EO (891.4 ± 262.7); NDL.EO (1021.4 ± 255.4); DL. EC (2982.3 ± 206.7); NDL. EC (2758.7 ± 568.9); Dul. FS. EO (1269.6 ± 490.2); ul. FS.EC (2696.9 ± 1098.3)
Ben Hassen et al. [68] *Purely Cross-Sectional*	Evaluate and compare jumps and accelerations based on playing position	GK: (*n* = 12) (17.3 ± 0.5 y)/Male	Explosive Strength (SJ (cm)); Linear Speed (Sprint 10 m, 20 m, 30 m)	SJ (29.2 ± 5.0; *p* < 0.05); S10(2.14 ± 0.08 s; *p* < 0.001); S20 (3.55 ± 0.12 s; *p* < 0.001); S30 (4.86 ± 0.18 s; *p* < 0.001)
Vagle et al. [69] *Purely Cross-Sectional*	Map anthropometric and physical performance profiles	GK: (*n* = 14) O19 (22 ± 4 y)/Male	Explosive Strength (CMJ (cm)); Linear Speed (Sprint 20, 30, 40 m); Speed with change in directions (COD) (shuttle run lineal with dominant and non-dominant legs (s))	Sprint 20 m (s) (3.21 ± 0.14); Sprint 30 m (s) (4.57 ± 0.20); Sprint 40 m (s) (5.92 ± 0.28); shuttle run dominant leg (s) (10.50 ± 0.39); CMJ (cm) (32.6 ± 4.5). Significant difference in Sprint 20, 30, 40 m and shuttle run performance between dominant and non-dominant legs (*p* < 0.01). Goalkeepers showed significantly worse shuttle run performance compared to field players (*p* < 0.01 for comparisons with midfielders, defenders, and attackers)
Baroni and Leal Junior [70] Purely *Cross-Sectional*	Assessing the anaerobic capacity of young football players	GK: (*n* = 3)/U16 (15.33 ± 0.58 y)/Male	Strength (Wingate (WanT) (W) (Peak power (PP), Average power (AP)) (W/kg) (Relative peak power (RPP), Relative average power (RAP)) and Fatigue Index (FI)(%))	PP = 737.57 ± 59.77 (W): RPP = 10.43 ± 0.47 W/kg; AP = 580.17 ± 52.61 W; RAP = 8.21 ± 0.49; FI = 41.32 ± 5.88%
Jadczak et al. [71] *Purely Cross-Sectional*	Compare balance profiles based on different field positions	GK: (*n* = 10) O19 (24.37 ± 4.53 y)/Male	Dynamic Stability ((Dom and Ndom); DPPT (Dynamic postural priority test (%)); ST (static balance; OE (eyes opened); CE (Eyes Closed (°))	DPPT (Dom = 48.79 ± 7.51%; Ndom = 5.58 ± 7.95%); ST OE = (Dom = 1.14 ± 0.62°; Ndom = 1.65 ± 1.35°); ST CE = (Dom = 4.04 ± 2.18°; Ndom = 3.02 ± 1.76°)
Ruas et al. [72] *Purely Cross-Sectional*	To compare isokinetic strength profiles in football players in different field positions	GK: (*n* = 12) O19 (26 ± 6 y)/Male	Strength (Isokinetic Dynamometer Test (N.M) (Quadriceps and Hamstrings peak torque test (QPT y HPT); Eccentric peak torque (EPT) (Dom, NDom, Asymmetry (%), Conventional and Functional ratio	QPT (Dom: 302 ± 34 y NDom: 294 ± 37); Asymmetry (%): +9 ± 4; HPT (Dom: 182 ± 35 y NDom: 162 ± 31); Asymmetry (%): +15 ± 13. EPT (Dom: 247 ± 54 y NDom: 211 ± 36); Asymmetry (%): +18 ± 12. Conventional ratio (Dom: 0.60 ± 0.07 y Ndom: 0.55 ± 0.08) Functional ratio (Dom: 0.81 ± 0.09 y Ndom: 0.72 ± 0.10)
Tsiokanos et al. [73] *Purely Cross-Sectional*	To compare the isokinetic peak torque of the knee extensors in relation to their playing position on the field	GK: (*n* = 24) O19 (28.3 ± 2.9 y)/Male	Strength (Isokinetic Dynamometer (Peak torque (PT) (Nm) (30–60–180°/s). F/S ratio 180/30 (0.56 ± 0.04): Peak torque/body weight (Nm/kgf) (30–60–180°/s)))	PT (Nm) (30°/s) = 355 ± 45; (60°/s) = 312 ± 44; (180°/s) = 198 ± 30. F/S ratio 180/30 (0.56 ± 0.04): Peak torque/body weight (Nm/kgf) (30°/s) = 4.3 ± 0.4; (60°/s) = 3.7 ± 0.4; (180°/s) = 2.4 ± 0.3
Charneco Salguero et al. [74] *Purely Cross-Sectional*	Evaluation of the isokinetic muscle profile of the knee extensors and flexors and comparison of asymmetries and between positions on the field	GK: (*n* = 32)/O19 (21.7 ± 4.6 y)/Male	Strength (Isokinetic Dynamometer (Peak torque (PT) (extension and flexion of (right (R) and left leg (L)) at (60°, 180°, and 240° (Nm)) and its average (Av) work on each leg at 60°, 180°, and 240° (J)))	Extension PT 60 (Nm) (R (245.47 ± 46.15); L (246.25 ± 38.32 a)): 180 (Nm) (R (195.07 ± 23.56 b); L (192.73 ± 27.88 d)): 240 (Nm) (R (162.47 ± 20.32); L (160.60 ± 23.25 e)). Flexion PT 60 (Nm) (R (143.66 ± 20.30); L (129.13 ± 27.71)): 180 (Nm) (R (119.33 ± 19.04); L (106.23 ± 22.7)): 240 (Nm) (R (107.13 ± 19.93), L (95.37 ± 20.02)). Av Extension = (60 (J) (R (242.20 ± 41.64); L (224.01 ± 63.91)): 180 (J) (R (188.25 ± 28.90 a); L (187.31 ± 30.95)): 240 (J) (R (119.72 ± 17.01); L (119.08 ± 17.37)). Av Flexion work 60 (J) (R (156.39 ± 29.71); L (144.17 ± 30.86)): 180 (J) (R (119.80 ± 19.83); L (106.79 ± 24.96)): 240 (J) (R (76.93 ± 17.09); L (66.34 ± 17.62))
Germano et al. [75] *Purely Cross-Sectional*	Evaluation of the isokinetic muscle profile of the knee extensors and flexors and comparison of asymmetries between field positions	GK: (*n* = 16) O19 (26.21 ± 7.07 y)/Male	Strength (Isokinetic Dynamometer (knee extensors (Kex) and flexors (Kfl) at 60° and 240° in dominant (Dom) and non-dominant (NDom) (AngPT: Peak torque angle (°); TPT: Peak torque time (in milliseconds (ms)) AcT: Acceleration time (in milliseconds); and PT (Peak torque))	240° DOMINANT LIMB: AngPT (Kex (64 ± 13.75°), Kfl (77 ± 27°)); TPT (Kex (120 ± 70 ms), Kfl (330 ± 117.5 ms)). AcT; Kex (40 ± 10 ms), Kfl (77.5 ± 43.25 ms). 240° NON-DOMINANT LIMB: AngPT: Kex (155 ± 65°), Kfl (340 ± 192.5°). TPT: Kex (120 ± 70), Kfl (330 ± 117.5). AcT: Kex (40 ± 17.5), Kfl (70± 27.5). 60° DOMINANT LIMB: AngPT: Kex (Dom: 64.21 ± 9.64°; NDom:60.14 ± 8.57°) Kfl (Dom: 417.85 ± 168.94°; NDom: 470 ± 136.21°). TPT: Kex (Dom: 417.85 ± 168.94 ms; NDom: 60.14 ± 8.57 ms), Kfl (Dom: 397.14 ± 156.61 ms; NDom: 414.28 ± 116.4 ms). AcT: Kex (Dom: 30.71 ± 13.28 ms; NDom: 24.28 ± 9.37 ms), Kfl (Dom: 34.28 ± 12.22 ms; NDom: 33.57 ± 10.08 ms): PT = Kex (Dom: 282.72 ± 51.35 ms; NDom: 283.36 ± 41.74 ms), Kfl (Dom: 160.59 ± 17.23 ms; NDom: 154.63 ± 25.24 ms)

Y = years; m = metres; cm = centimetres; s = seconds; ms = milliseconds; W = watts; J = Joule; ° = Degrees; N = Newton; Kg = kilograms; BW = body weight; U14 = Under 14 years; U16 = Under 16 years; U19 = Under 19 years; O19 = Over 19 years; GK = Goalkeeper; Starting GK = Main GK plays more games; Backup GK = substitute goalkeeper, plays fewer games; * = *p* < 0.05; ** = *p* < 0.01; ^†, ‡, §^ = The labels indicate significant differences among the five positions; SJ = Squat jump; CMJ = Countermovement jump; VJ = Vertical jump; SH = Horizontal jump; Reaction and Action Speed (RAS); S10 = Sprint 10 m; S20 = Sprint 20 m; SR = Sit and reach; MSR = Modified sit and reach; WanT = Wingate; Δfmax = Force asymmetry; Kex = Knee extensors; Kfl = Flexors; L (Left); R (Right); Dom = Dominant; Ndom = Non-dominant; QPT = Quadriceps peak torque test; HPT = Hamstrings peak torque; EPT = Eccentric peak torque; Fmax = Maximal Force; Vmax = maximal velocity; P = power; AP = Absolute power; RP = Relative power; PP = Peak power; MP = Mean power; RPP = Relative peak power; RMP = Relative mean power; PT = Peak torque; AngPT = Peak torque angle; TPT = Peak torque time; AcT = Acceleration time; RA = Reach Asymmetry; ATT = Arrowhead Agility Test; DF = Deficit Balance; DSR-R = Dominant straight leg raise; NDSR-R = Dominant straight leg raise; PL = Path length; YBT = Y-Balance Test; DL EO = Dominant Leg, Eyes Open; NDL.EO = Non-dominant Leg; DL. EC = Dominant Leg, Eyes Closed; NDL.EC = Non-dominant Leg, Eyes Closed; Dul. FS. EO = Double Leg Foam Surface Eyes Open; Dul. FS.EC = Double Leg Foam Surface Eyes Close; DPPT = Dynamic postural priority test; ST OE = static balance with eyes opened; ST CE = static balance with eyes opened; ROM = range of movement; COD = change in direction; FI = Fatigue Index; PHF KF = Hip flexion with knee flexed; PHF KE = Hip flexion with knee extended; PHE = Hip extension; PHA = Hip abduction; PHIR = Hip internal rotation; PHER = Hip external rotation; PKF = Knee flexion; ADFKF = Ankle dorsiflexion with knee flexed; ADF KE = Ankle dorsiflexion with knee extended; SLVJ = Single Leg Vertical Jump; SEBT = Star excursion balance test, SLJ = Standing Long Jump, A505Dom = agility 505 dominant, A505ND = Non-dominant.
